# Non-pathogenic *Escherichia coli* biofilms: effects of growth conditions and surface properties on structure and curli gene expression

**DOI:** 10.1007/s00203-020-01864-5

**Published:** 2020-03-28

**Authors:** James Leech, Stacey Golub, Wendy Allan, Mark J. H. Simmons, Tim W. Overton

**Affiliations:** 1 School of Chemical Engineering, College of Engineering and Physical Sciences, University of Birmingham, Edgbaston, Birmingham, B15 2TT UK; 2 Institute of Microbiology and Infection, University of Birmingham, Edgbaston, Birmingham, B15 2TT UK; 3 Present Address: School of Biosciences, University of Kent, Canterbury, Kent, CT2 7NH UK

**Keywords:** Confocal laser scanning microscopy, Osmolarity, Biofilm, Curli

## Abstract

Biofilm formation is a harmful phenomenon in many areas, such as in industry and clinically, but offers advantages in the field of biocatalysis for the generation of robust biocatalytic platforms. In this work, we optimised growth conditions for the production of *Escherichia coli* biofilms by three strains (PHL644, a K-12 derivative with enhanced expression of the adhesin curli; the commercially-used strain BL21; and the probiotic Nissle 1917) on a variety of surfaces (plastics, stainless steel and PTFE). *E. coli* PHL644 and PTFE were chosen as optimal strain and substratum, respectively, and conditions (including medium, temperature, and glucose concentration) for biofilm growth were determined. Finally, the impact of these growth conditions on expression of the curli genes was determined using flow cytometry for planktonic and sedimented cells. We reveal new insights into the formation of biofilms and expression of curli in *E. coli* K-12 in response to environmental conditions.

**Electronic supplementary material:**

The online version of this article (10.1007/s00203-020-01864-5) contains supplementary material, which is available to authorized users.

## Introduction

Biofilms represent a ubiquitous mode of growth for many microbes (Donlan 2002). Bacteria attach to solid surfaces in first a reversible, then an irreversible, manner; bacteria then multiply, forming microcolonies, and synthesise extracellular polymeric substances (EPS), generating a matrix and providing structure to the biofilm. These molecular determinants of each phase of biofilm development are tightly regulated in response to environmental stimuli and are highly species- and strain-specific. Often, biofilm formation is triggered as a result of stressful environmental conditions. Bacteria in biofilms are frequently more resistant to chemical and physical stresses (Koo et al. 2017) and antibiotics (reviewed by (Olsen 2015)) than planktonic bacteria and, therefore, represent a clinical problem in indwelling medical devices (Arciola et al. 2018). In addition, biofilms are resistant to cleaning in industrial settings such as industrial food processing (Coughlan et al. 2016) and on the underside of ships (Banerjee et al. 2011).

The robust nature of biofilms that makes their removal difficult in clinical and industrial settings makes them a potentially advantageous platform for biocatalysis (Rosche et al. 2009; Winn et al. 2012). For example, *Pseudomonas taiwanensis* biofilms efficiently catalyse the epoxidation of styrene to (S)-styrene oxide (Karande et al. 2014; Schmutzler et al. 2017). *Escherichia coli* strains K-12 and B are very commonly-used in biotechnology for biocatalysis and recombinant protein production (Overton 2014). They are non-pathogenic, have a long history of safe use and are well-characterised. Recent work has revealed that *E. coli* biofilms can effectively convert 5-haloindoles and l-serine to l-5-halotryptophan, catalysed by recombinant *Salmonella enterica* tryptophan synthase TrpBA (Tsoligkas et al. 2011). Formation of *E. coli* biofilms using spin-coating methods was found to rapidly generate robust biocatalytic biofilms (Tsoligkas et al. 2012); subsequent work focused on optimising the tryptophan synthase reaction (Perni et al. 2013) and revealed that enzyme turnover was at least partially responsible for the improved biocatalytic potential of biofilms (Tong et al. 2016).

Biofilm formation in *E. coli* K-12 is driven by a number of biological determinants (Beloin et al. 2008). Initial attachment in *E. coli* K-12 strains is primarily mediated by the curli, fimbrae of several micrometres in length projecting from the cell surface (Vidal et al. 1998; Olsen 2015; Van Gerven et al. 2015). Bacterial cohesion is mediated by the autoagglutanin Ag43 (Danese et al. 2000a). Flagella have also been shown to be involved in biofilm formation (Pratt and Kolter 1998). Later stages of biofilm development involve the production of polysaccharide EPS components (Limoli et al. 2015): poly-*N*-acetylglucosamine (PNAG), synthesised by products of the *pgaABCD* operon (Cerca and Jefferson 2008); colanic acid (a branched polymer comprising fucose, galactose, glucuronic acid and glucose, synthesised by the *wca* operon products) (Danese et al. 2000b); and cellulose (poly-β-1,4-glucose), generated by products of the *yhjR-bcsQABZC* and *bcsEFG* operons. *E. coli* K-12 strains possess a deletion in the *bcsQ* gene, resulting in a lack of cellulose production (Serra et al. 2013). Biofilm structure and function is thereby specified by the relative production of these determinants, which is subject to a complex regulatory network integrating multiple environmental stimuli (Domka et al. 2007).

In this study, we investigated the generation of biofilms by three non-pathogenic *E. coli* strains: K-12, represented here by strain PHL644, which contains a mutation in *ompR* increasing curli expression (Vidal et al. 1998); the industrially-used BL21 (an *E. coli* B strain); and the human probiotic Nissle 1917 (Wassenaar 2016). We studied and optimised biofilm formation on different surfaces and under different growth conditions. Biofilm formation differs in different *E. coli* strains, shown both in the literature (for example, (Schiebel et al. 2017)) and in this study, therefore, such studies on different strains are important. In addition, many studies focus on pathogenic *E. coli* strains, thus studies on non-pathogenic strains offer an interesting comparison. Finally, we investigated the effect of growth medium components and growth conditions on curli gene expression in PHL644. We show that regulation of curli expression is a primary driver of biofilm formation in *E. coli* K-12, with conditions giving rise to high biofilm formation also promoting curli expression. We also investigated the influence of osmolarity on the differences in curli expression observed in planktonic and sedimented bacteria, and reveal that osmolarity is likely to play a key role in the switch from a planktonic to a sessile lifestyle.

## Materials and methods

### Bacteria strains and plasmids

*Escherichia coli* K-12 strain PHL644 (*araD139 *a*(argF-lac)U169 rpsL150 relA1 flbB5301 deoC1 ptsF25 rbsR malA-kan ompR234* (Vidal et al. 1998)) was used. BL21 star (DE3) (F^−^*ompT hsdS*_B_ (r_B_^−^, m_B_^−^) *gal dcm rne*131 (DE3)) was sourced from Invitrogen (Paisley, UK). *Escherichia coli* Nissle 1917 was sourced from Mutaflor (Herdecke, Germany). Reporter plasmid pJLC-T comprises the *E. coli* MC4100 *csgD-csgB* intergenic region upstream of the gene encoding eGFP with a C-terminal AANDENYALVA tag which reduces GFP half-life to around 60 min (Andersen et al. 1998) cloned into the *Eco*RI-*Hin*dIII sites of pPROBE’-TT upstream of the *gfp* gene (Miller et al. 2000). pPROBE’-TT encodes tetracycline resistance and has a pBBR1 origin of replication.

### Biofilm growth media

Unless otherwise stated, antibiotics were omitted from biofilm growth media to prevent effects on biofilm formation. Standard M63 minimal medium contained 100 mM KH_2_PO_4_, 15 mM (NH_4_)_2_SO_4_, 1 mM MgSO_4_, 1.8 µM FeSO_4_ and 10 mM D-glucose. M63+ medium comprised M63 medium with the addition of 17 mM sodium succinate. LB medium contained 10 g L^−1^ tryptone (Sigma-Aldrich, Poole, UK), 5 g L^−1^ yeast extract (Oxoid, Basingstoke, UK) and 10 g L^−1^ NaCl (ThermoFisher, Paisley, UK).

### Coupon preparation

Glass coverslips (SLS, Nottingham, UK) were cleaned with ethanol, dried, then autoclaved to sterilise. Polystyrene (PS) coupons were obtained by cutting square Petri dishes (SLS) with a scalpel into 22 × 32 mm coupons. Polycarbonate (PC) sheets were purchased (RS Components, Corby, UK) and cut into 22 × 32 mm coupons. PS and PC coupons were washed and soaked in ethanol for > 2 h to sterilise, then dried before insertion into 6-Well Plates. 304-grade stainless steel (SS) coupons (22 mm × 32 mm) were cut from sheet material (University of Birmingham workshops), washed with acetone and ethanol, dried, then autoclaved to sterilise. After use in biofilm experiments, SS coupons were washed thoroughly with acetone, ethanol and deionised water and reused. Glass microscope slides (75 mm × 25 mm; ThermoFisher) were washed with ethanol, dried, and sterilised by autoclaving. PTFE-wrapped microscope slides were prepared by wrapping PTFE tape (RS Components) around a glass microscope slide to cover the entire surface. These were washed with deionised water, dried, and then sterilised by autoclaving.

### Biofilm growth methods

**Fig. 1:**
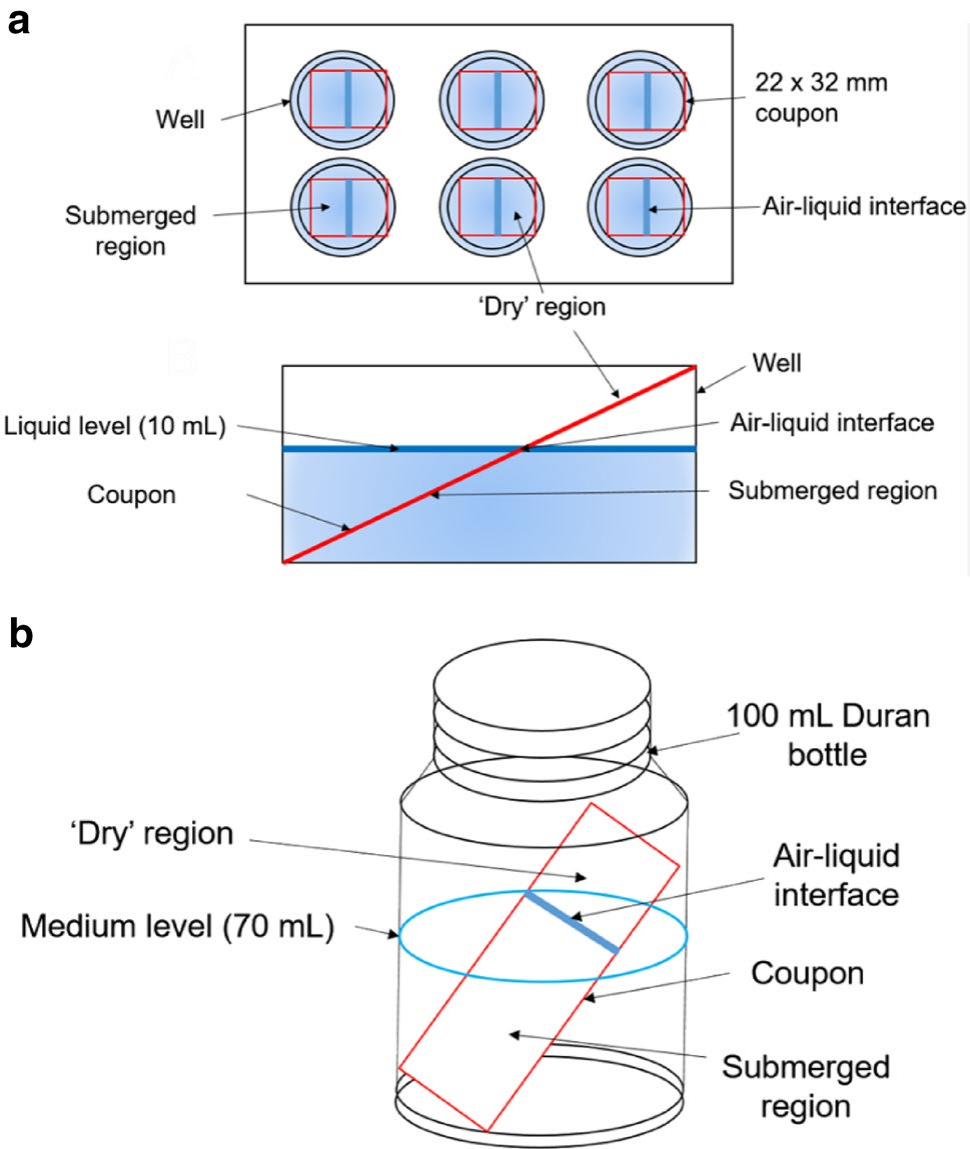
Schematic of **a** 6-well plate and **b** Duran bottle methods of biofilm formation

For the 6 well plate method, a 22 × 32 mm coupon was inserted at an angle into each well of a sterile polystyrene 6-well microplate (ThermoFisher) to which was added 10 mL of growth medium and a 100 µL of overnight culture (Fig. 1). For the Duran bottle method, a polyurethane bung was inserted into a 100 mL Duran Bottle and sterilised. Medium and a 1 in 100 dilutions of the overnight culture was added to the Duran Bottle to a final volume of 70 mL, and a sterile slide was inserted into the bottle. Plates or Duran bottles were incubated in a MaxQ 4000 orbital shaker (Thermo Scientific, Paisley, UK) with an orbit of 19 mm at 30 °C and 70 rpm for 3 days.

### Crystal violet staining

For 6-well plates, 10 µL of 1% Crystal Violet (CV) solution (Sigma-Aldrich) was added to each well of the 6-Well Plate and left to stain for 1 h at 30 °C and 70 rpm. The concentration of CV in each well was 0.001%. Stained coupons were then removed with tweezers and washed three times by gentle submersion in PBS to remove non-adhered cells. The coupons were then inserted into a new, clean 6-well Plate with 10 mL of 95% ethanol (ThermoFisher) in each well for 30 min at room temperature and 70 rpm to destain. The coupons were discarded and the OD_580_ of the destain solution was measured using an Evolution 300 UV–Vis spectrophotometer (Thermo Fisher Scientific, Hemel Hempstead, Herts, UK) controlled by Thermo Scientific™ VISIONpro™ software. For Duran bottles, 70 µL of 1% CV solution was added directly to each bottle and left to stain at 30 °C and 70 rpm for 1 h. The CV concentration in each bottle was 0.001%. The stained slide was removed and washed three times by gentle submersion in PBS to remove non-adhered cells. Each stained slide was inserted into a fresh 50 mL centrifuge tube with 35 mL of 95% ethanol for 30 min to destain. The slides were discarded and the OD_580_ of the destain solution was measured using a spectrophotometer. The blank in each case was a cell-free control (medium-plus coupon or slide, respectively). Data were tested for significance using a paired two-tailed *T*-test (Microsoft Excel).

### Confocal laser scanning microscopy (CLSM)

A biofilm-coated coupon or slide was removed from a 6-well Plate or Duran Bottle and washed three times by submersion in PBS to remove non-adhered cells. The coupon was then placed biofilm-side-up onto a clean microscope slide. 20 µL of 200 µM SYTO62 (ThermoFisher) was applied to the biofilm-coated surface of the coupon and left to stain in dark conditions for 15 min. A 22 mm × 32 mm glass coverslip was gently applied to the top of the area to be viewed, then the sample was imaged using a Leica TCS SPE CLSM with a 40 × oil immersion lens. SYTO62 was excited by a 635 nm laser and emission was detected in the range of 670–690 nm.

### Gene expression studies

A polyurethane bung was inserted into a 100 mL Duran Bottle and sterilised. Medium and a 1 in 100 dilution of the overnight culture were added to the Duran Bottle to a final volume of 70 mL. Bottles were incubated in a MaxQ 4000 orbital shaker (Thermo Scientific) with an orbit of 19 mm at 30 °C and 70 rpm for 3 days. One millilitre samples were taken at regular time points and analysed by spectrophotometry (OD_600_) and flow cytometry. For flow cytometry, cells were diluted in PBS buffer (Sigma) and immediately analysed using an Accuri C6 (BD Biosciences, Wokingham, UK) with excitation at 488 nm. Green fluorescence was collected through a 533/30 bandpass filter. Mean FL1-A values are reported.

### Goiniometry

Goiniometry (contact angle determination) was used to measure the relative hydrophobicity of materials used in this study using a home-built contact angle apparatus as described by (Stephenson-Brown et al. 2013). A small drop of deionised water was dripped onto each material coupon from a hypodermic needle suspended approximately 20 cm above the coupon. An image of the droplet on the surface was obtained and the angle at which it contacted the surface was measured using ImageJ (Schneider et al. 2012). All measurements were performed three times with independent coupons.

## Results and discussion

There are numerous growth models for biofilm formation as recently reviewed by (Azeredo et al. 2017). Our previous work on biocatalytic *E. coli* biofilms (Tsoligkas et al. 2011, 2012; Perni et al. 2013) used a spin-coating method followed by a period of maturation to generate robust biofilms capable of effective biocatalysis. In this study, to complement the spin-coating methods, we developed methods of biofilm growth that relied upon natural biofilm formation by non-pathogenic *E. coli* strains. Two growth methods were developed (Fig. 1); a 6-well plate method, growing bacteria on 22 × 32 mm coupons (Fig. 1a); and a larger-scale, Duran bottle method, growing bacteria on microscope slides (Fig. 1b). In both methods, the surface on which the biofilm grew was angled, as preliminary experiments revealed that an angled surface enabled better biofilm formation than a surface flat on the bottom of a well plate or bottle.

**Fig. 2:**
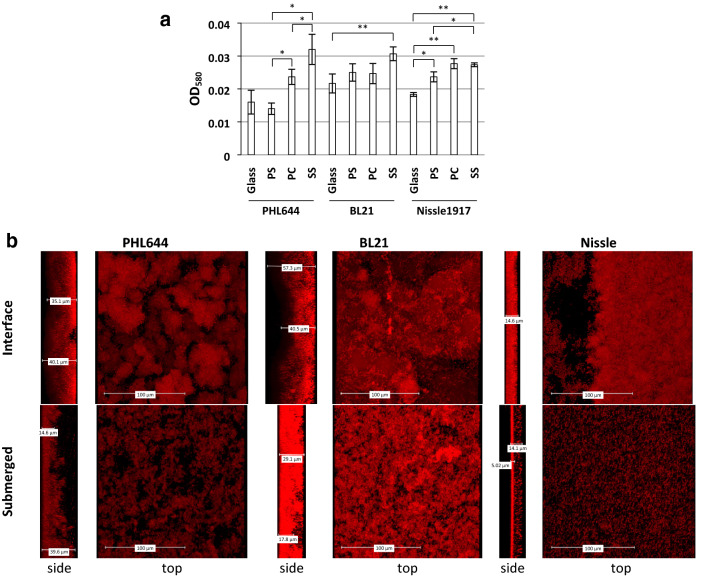
Comparison of biofilm formation by three strains (PHL644, BL21 and Nissle 1917) on different substrates. The 6-well plate method was used to compare biofilm formation in M63+ medium on glass, polystyrene (PS), polycarbonate (PC) and stainless steel (SS) coupons. After 3 days, crystal violet staining was used to quantify biofilm formation (**a**). Data shown are the mean ± standard deviation of 3 independent coupons. Significance was determined using a paired two-tailed *T*-test: *p < 0.05, **p < 0.01. CLSM was also used to image the biofilm grown on glass coupons stained with SYTO62 (**b**); representative side- and top-view images are shown, side views show the base of the biofilm on the left and the top of the biofilm on the right

Initial experiments investigated biofilm formation on a range of abiotic materials. Three non-pathogenic strains of *E. coli* were used: PHL644, a K-12 derivative with an *ompR234* allele conferring overexpression of the adhesin curli via upregulation of the curli master regulator CsgD (Vidal et al. 1998); BL21 star (DE3), referred to here as BL21, a B strain commonly used for recombinant protein production (Invitrogen); and the probiotic Nissle 1917, which has been previously reported to form biofilms well (Hancock et al. 2010). First, each strain was grown for 3 days on coupons of different materials that are used industrially and have previously been reported to support biofilm growth (glass, polystyrene (PS), polycarbonate (PC) and stainless steel (SS)) at 30 °C using the 6 well plate method (Fig. 2). Crystal violet was used to quantify biofilm formation (Fig. 2a); each strain tested formed biofilms on each substratum. Strain PHL644 generated similar quantities of biofilm on glass and PS, with more biofilm formation on PC and SS. Strain BL21 formed equivalent quantities of biofilm on glass, PS and PC and more on SS; Nissle 1917 generated the most biofilm on PC and SS.

Confocal laser scanning microscopy (CLSM) was used to visualise the biofilms following staining with the permeant DNA dye SYTO62 (Fig. 2b). In the 6 well plate model, visually distinct biofilms grew on coupons submerged beneath the surface of the liquid and at the air–liquid interface. Therefore, both submerged and interface biofilms were imaged using CLSM (Fig. 2b). Interface biofilms tended to be thicker (ie have a greater depth) and denser than submerged biofilms. For strain PHL644, the interface biofilm was denser and more uniformly thick than the submerged biofilm, although maximum submerged thickness was comparable to interface thickness at around 40 µm. BL21 generated a thicker interface biofilm than PHL644 (50 µm), but a thinner submerged biofilm (around 30 µm); however, as the cells in the submerged biofilm were more densely packed than those in the interface biofilm, overall cell numbers were probably comparable. Overall morphology of PHL644 and BL21 biofilms were similar. *E. coli* Nissle formed thinner biofilms than PHL644 and BL21, suggesting that these growth conditions were suboptimal for Nissle biofilm formation.

**Fig. 3:**
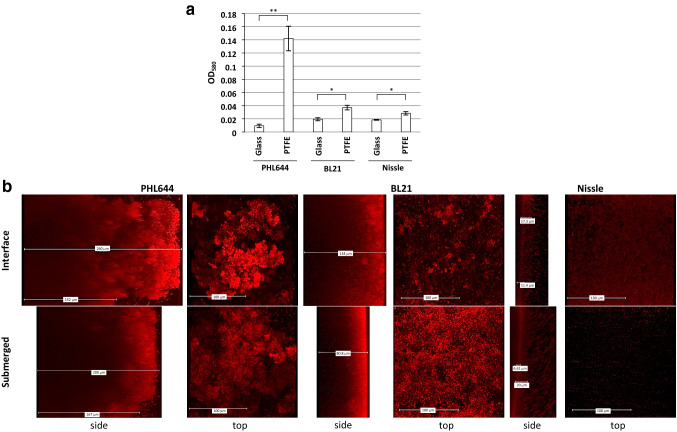
Biofilm formation on PTFE. The Duran bottle method was used to compare biofilm formation in M63+ medium on glass and PTFE. After 3 days, crystal violet staining was used to quantify biofilm formation (**a**). Data shown are the mean ± standard deviation of 3 independent coupons. Significance was determined using a paired two-tailed *T*-test: *p < 0.05; **p < 0.01. CLSM was also used to image the biofilm grown on PTFE stained with SYTO62 (b); representative side- and top-view images are shown, side views show the base of the biofilm on the left and the top of the biofilm on the right

To improve biofilm formation, polytetrafluoroethene (PTFE) was tested as a surface for biofilm attachment. These experiments used the Duran bottle method (Fig. 1b) as PTFE-wrapped coverslips floated in the 6 well plate method. Crystal violet assays (Fig. 3a) revealed that all strains formed more biofilm on PFTE than glass microscope slides, with PHL644 forming the greatest quantity. CLSM analysis of biofilms grown on PTFE (Fig. 3b) revealed thicker biofilms formed by PHL644 and BL21 than on glass substrata, but similarly thin biofilms for Nissle. As with glass substrata, PHL644 and BL21 biofilms were thickest at the interface. PHL644 formed the thickest biofilms both at the interface (around 260 µm) and submerged (200 µm) locations.

Goniometry was used to quantify the hydrophobicity of each substratum tested (Table 1). PTFE was the most hydrophobic, followed by PC, PS and SS, and glass was the most hydrophilic surface. Substratum hydrophobicity has previously been shown to influence biofilm formation, although it is not the only factor important for attachment (Hook et al. 2012). Strains with the *ompR234* allele conferring increased curli expression (as PHL644 used here) were initially identified as having enhanced adhesion to hydrophobic plastic (PS) as well as hydrophilic glass surfaces (Vidal et al. 1998).

**Table 1:** Comparison of contact angles observed for deionised water droplets on materials tested in this study. Mean and standard deviation values were derived from 3 independent tests

Material	Mean contact angle (°)
Glass	43 ± 1.53
PS	85 ± 3.51
PC	84 ± 2.52
SS	89 ± 1.73
PTFE	113 ± 3.06

Some previous studies have shown that curli-producing *E. coli* O157 strains are more hydrophobic than their non-curli producing counterparts (Boyer et al. 2007; Patel et al. 2011), although other studies have found this not to be the case (Goulter et al. 2010); the area has been reviewed (Goulter et al. 2009), highlighting differences in strains, media and methods used. Biophysical analysis has revealed interactions between curli and hydrophobic surfaces (DeBenedictis et al. 2016) or in uropathogenic *E. coli* pellicles (biofilms floating at the air–liquid interface (Wu et al. 2012)).

### Optimising growth of PHL644 biofilms on PTFE

**Fig. 4:**
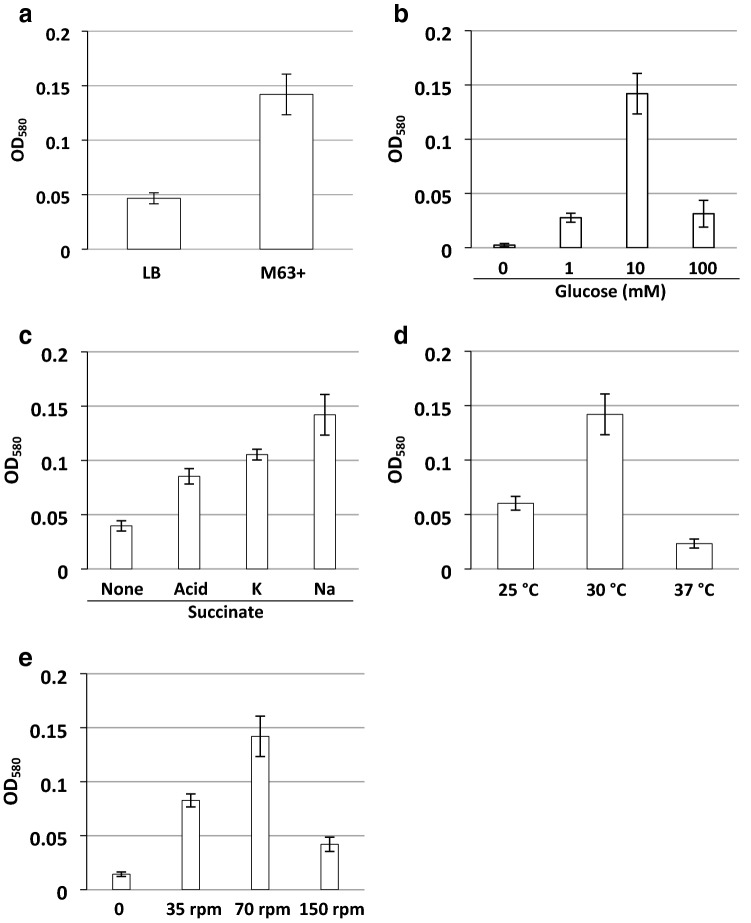
Optimisation of growth conditions for PHL644 biofilm formation on PTFE. Biofilms were grown on PTFE-wrapped glass slides under different conditions using the Duran bottle method. Biofilm formation was quantified after 72 h using crystal violet staining. **a** Rich medium (LB) and minimal medium (M63+) were compared. **b** Different concentrations of glucose in M63+ medium were compared. **c** Presence or absence of different succinate sources were compared. **d** Different temperatures were compared. **e** Different rotational speeds were compared. In each case, error bars represent 1 standard deviation from the mean of 3 independent coupons

Conditions were optimised to generate *E. coli* PHL644 biofilms on PTFE supports using the Duran bottle method. The effect of growth medium has previously been shown to have a large impact on *E. coli* biofilm formation in numerous studies (Pratt and Kolter 1998; Prigent-Combaret et al. 2000; Naves et al. 2008). Crystal violet analysis revealed PHL644 formed more biofilm on PTFE when grown in minimal M63+ medium than in rich LB medium (Fig. 4a). CLSM confirmed that biofilms grown in M63+ medium were thicker (> 200 µm) than those grown in LB (< 70 µm; data not shown). Glucose has previously been shown to repress biofilm formation via catabolite repression and CRP-cAMP (Jackson et al. 2002; Hufnagel et al. 2016). Here, CV analysis revealed that biofilm formation was greatest in the presence of 10 mM glucose (Fig. 4b). Higher glucose concentrations resulted in less biofilm formation (presumably via catabolite repression) whereas lower glucose concentrations would be likely to inhibit growth, limiting biofilm biomass.

In addition to glucose, succinate was used as a carbon source in M63+ medium (Tsoligkas et al. 2011, 2012). Sodium and potassium succinate were tested as sodium ions have previously been shown to influence biofilm formation, specifically via activation of the *pgaABCD* operon by NhaR (Goller et al. 2006). The effect of omission or replacement of sodium succinate with potassium succinate or succinic acid was tested (Fig. 4c); sodium succinate was shown to induce optimal biofilm growth. Omission of succinate did not prevent biofilm formation, unlike omission of glucose. Succinate can be used as a carbon source by *E. coli* (Engel et al. 1992), although in the presence of glucose it will be used as a secondary carbon source as the succinate importers DctA and DcuB are catabolite repressed (Lo et al. 1972; Golby et al. 1998).

Finally, incubation temperature and rotational speed were optimised. Temperature is known to have a significant effect on *E. coli* biofilm formation (Barnhart and Chapman 2006; Rühs et al. 2013; Uhlich et al. 2014); curli expression is regulated by the thermosensitive Crl protein via RpoS (Bougdour et al. 2004; Brombacher et al. 2006). 30 °C was confirmed to be the optimal temperature for growth of PHL644 biofilms here (Fig. 4d). Biofilm formation was found to be optimal at a rotational speed of 70 rpm (Fig. 4e); higher or lower rotation decreased biofilm formation. The effect of shear on biofilm formation has previously been investigated. *P. aeruginosa* biofilm coverage in a flowcell was found to increase with shear stress up to a limit, then decrease upon additional increases in shear stress (Park et al. 2011). *E. coli* is also known to attach to mannosylated surfaces via FimH fimbrae which display catch bond characteristics; the adhesion is enhanced by shear forces (Thomas et al. 2004). However, it is not known if FimH has a role in adhesion to PTFE, or if curli exhibit catch bond character. Alternatively, rotation may enhance mixing in the Duran bottles and enable bacteria to be passively transported to the PTFE surface for attachment to occur.

### Analysis of curli gene expression

Given that initial attachment to abiotic surfaces in *E. coli* K-12 is primarily mediated by the adhesin curli, and we have shown that biofilm formation in this experimental system was enhanced under conditions that are known to result in increased curli expression (30 °C, low glucose concentration, minimal medium (Barnhart and Chapman 2006)), a reporter plasmid (pJLC-T) was constructed to allow measurement of *csgB* promoter activity. The *csgBAC* operon encodes the structural curlin genes. The *csgB* promoter region was fused to *gfp*, enabling analysis by flow cytometry (FCM).

**Fig. 5:**
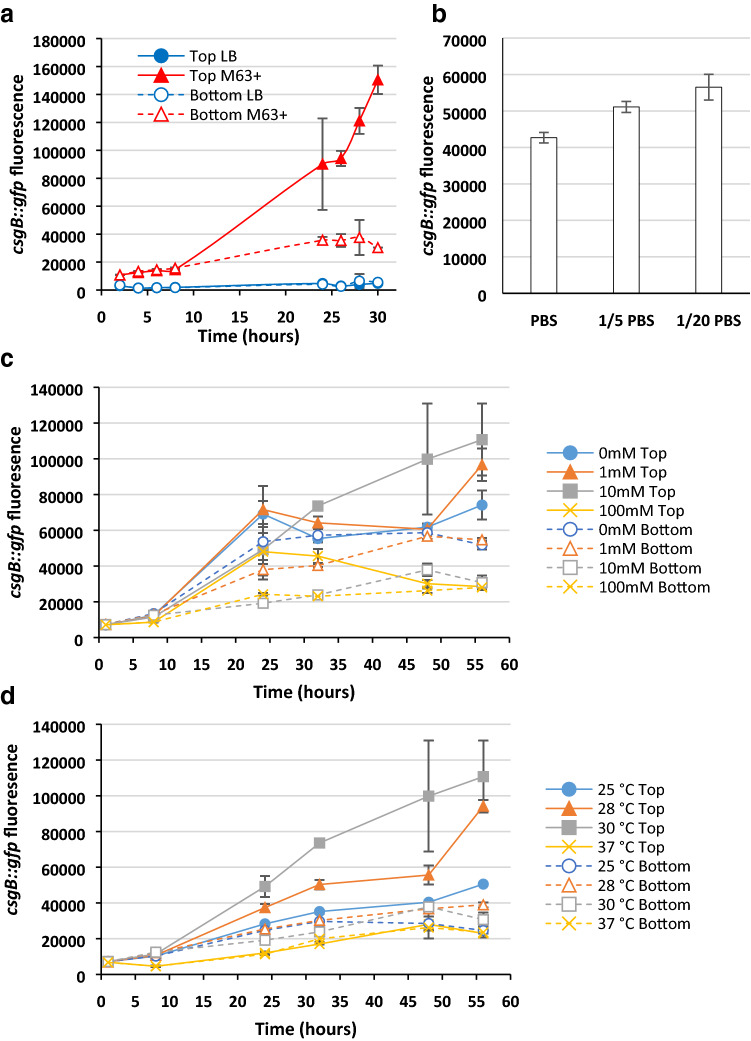
Expression of *csgB* in different growth conditions. PHL644 pJLC-T (carrying *csgB::gfp*) was grown in Duran bottles containing 70 mL of medium, 1 mL samples were taken from the top (planktonic cells) and bottom (sediment) and the green fluorescence measured by FCM. Cultures were grown in: **a** M63+ medium or LB medium, **c** M63+ medium containing 0, 1, 10 or 100 mM glucose, and **d** M63+ medium at 25 °C, 28 °C, 30 °C or 37 °C. Data shown are the mean ± standard deviation of the mean green fluorescence value (FL1-A) determined by flow cytometry of three independent cultures. Fluorescence values are stated in arbitrary units as reported by the BD Accuri C6 flow cytometer. In addition, the effect of osmolarity on resting PHL644 pJLC-T was determined (**b**): PHL644 pJLC-T cells were grown in M63+ medium at 30 °C overnight, 1 mL of sediment cells were taken and resuspended in 5 mL of PBS, 1/5 strength PBS or 1/20 strength PBS at a final biomass concentration of OD_600_ ≈ 0.08. After 24 h at 30 °C, green fluorescence was measured by FCM

First, we determined the activity of the *csgB* promoter during the growth of PHL644 in M63+ or LB medium. Transformants were grown as before in Duran bottles at 30 °C and 70 rpm shaking; samples were taken from the top and bottom of the bottle over time, allowing comparison of *csgB* expression in planktonic and sedimented cells, and green fluorescence was measured using FCM. From the start of growth, the fluorescence of the *csgB::gfp* fusion was higher in cultures grown in minimal M63+ medium than in rich LB medium (Fig. 5a), corresponding to crystal violet biofilm quantity data (Fig. 4a). This is similar to reporter gene data from previous studies for *E. coli* overproducing CsgD grown in minimal and rich media (Brombacher et al. 2006).

In addition, for cultures grown in M63+ medium, samples taken from the top of the bottle (planktonic cells) had higher fluorescence than those taken from the bottom (sediment) after 8 h growth; this effect was not observed in cells grown in LB medium, where fluorescence was similarly very low for both planktonic and sedimented cells throughout the experiment. Individual flow cytometry histograms for each sample (Supplemental Fig. S1) reveal that cultures grown in M63+ medium displayed a shift from two populations in respect to green fluorescence at early timepoints to a single population at late timepoints, albeit with the appearance of a second subpopulation in sediment samples at 28 h. Cultures grown in LB medium were far more heterogenous in terms of green fluorescence, having a major population with low fluorescence and a shoulder or second population having higher fluorescence. We hypothesise that the difference observed in M63+ medium is due in part to inhibition of curli expression by two factors: the higher osmolarity experienced by cells in sediment as a result of entrained solutes (Tuson and Weibel 2013); and communication between cells within the sediment, either chemical (for example quorum sensing) or mechanical (i.e. contact dependence). Curli production has previously been shown to be repressed in response to increased osmolarity, both in wild-type OmpR (Olsén et al. 1993) and *ompR234* backgrounds (Prigent-Combaret et al. 2001). To further investigate the relationship between osmolarity and *csgB* expression, we grew PHL644 pJLC-T in M63+ medium overnight, took 1 mL of sediment cells and resuspended them in 5 mL of either PBS, 1/5 strength PBS or 1/20 strength PBS at a final biomass concentration of OD_600_ ≈ 0.08. After 24 h static incubation at 30 °C, cultures were mixed and sampled; there was no increase in biomass concentration, but green fluorescence was inversely proportional to osmolarity (Fig. 5b). However, no such trend was observed when we repeated the experiment at a higher biomass concentration (OD_600_ of between 0.6 and 1). This suggests that both osmolarity and biomass concentration play a role in curli regulation during sedimentation, potentially through bacteria sensing the presence of other bacteria, and will be the focus of future studies.

Next, the effect of glucose concentration on *csgB* expression was determined by supplementing M63+ medium with either 0 mM, 1 mM, 10 mM (the standard concentration in M63+ medium) or 100 mM glucose (Fig. 5c). Curli expression in planktonic cells after 30 h was highest in the presence of 10 mM glucose; this observation corresponds with crystal violet data (Fig. 4b). 100 mM glucose led to the lowest planktonic curli expression. The difference between curli expression in planktonic and sediment cells was highest for 10 mM glucose, whereas expression in planktonic and sediment cells without addition of glucose were similar. Flow cytometry histograms (Supplemental Fig. S1) showed single populations for samples taken 24 h and later; from 1 to 8 h there was a shift from a low-fluorescence to a high-fluorescence population. Catabolite repression of curli production is mediated by the regulators CRP and CsgD; CRP binds to the *csgD* promoter (Zheng et al. 2004), activating expression of CsgD, the main activator of *csgB* (Hufnagel et al. 2016).

Finally, the effect of temperature on *csgB* expression was determined (Fig. 5d). In planktonic cells, expression was highest at 30 °C, reflecting biofilm biomass data (Fig. 4d). Expression in planktonic samples followed the order 30 °C > 28 °C > 25 °C > 37 °C. This confirms previous reporter data (Brombacher et al. 2006; Perrin et al. 2009). The thermosensitive Crl protein has previously been found to upregulate *csgB* expression at 30 °C by recruitment of the sigma factor σ^S^ (Bougdour et al. 2004; Brombacher et al. 2006). As before, curli expression in sediment cells was lower than for planktonic cells at each temperature, except at 37 °C where expression was similar in each location. The differences between *csgB* expression in sediment cells grown at different temperatures were also far smaller than those observed in planktonic cells, suggesting that thermoregulation of curli expression might be more important planktonically, or signals that downregulate curli expression in sediment override temperature-dependent regulation. Flow cytometry histograms (Supplemental Fig. S1) show similar patterns at 25–30 °C, with single populations and the tendency for a low-fluorescence shoulder in sediment samples. Samples grown at 37 °C showed a different pattern, with two populations in most samples and timepoints.

## Conclusions

*E. coli* biofilms were shown to form on a variety of surfaces, with PTFE identified as the preferred substratum. Strain PHL644 was the best biofilm former in the conditions tested and conditions for optimal biofilm formation were identified in terms of the growth medium, temperature, glucose concentration, succinate source and rotational speed during culture. Differences in biofilm formation in the different strains tested here reinforce the fact that different *E. coli* strains form biofilms in different ways and in response to different stimuli (Schiebel et al. 2017). Curli gene expression was shown to correlate to biofilm formation, reinforcing the function of curli as the initial adhesin of *E. coli* K-12.

Curli expression appears to be lower in sedimented cells than in planktonic cells, suggesting that planktonic cells are physically more capable of attaching to surfaces; when in a sediment, curli expression appears to be downregulated. Although osmolarity was shown to regulate curli expression, the switch from a planktonic to a sessile mode of growth is influenced by multiple factors including osmolarity and cell density, and the interaction of these factors requires further study.

## Electronic supplementary material

Below is the link to the electronic supplementary material.

Supplementary file1 (DOCX 4563 kb)
