# Supplementary material for: Non-pathogenic Escherichia coli biofilms: effects of growth conditions and surface properties on structure and curli gene expression
Source: Arch Microbiol. 2020 Mar 28;202(6):1517–27. doi: 10.1007/s00203-020-01864-5 (PMC7355273; doi:10.1007/s00203-020-01864-5)

**Supplemental Figure S1**

Flow cytometry green fluorescence histograms corresponding to Figure 5.

#

30h

28h

26h

24h

4h

2h

a)


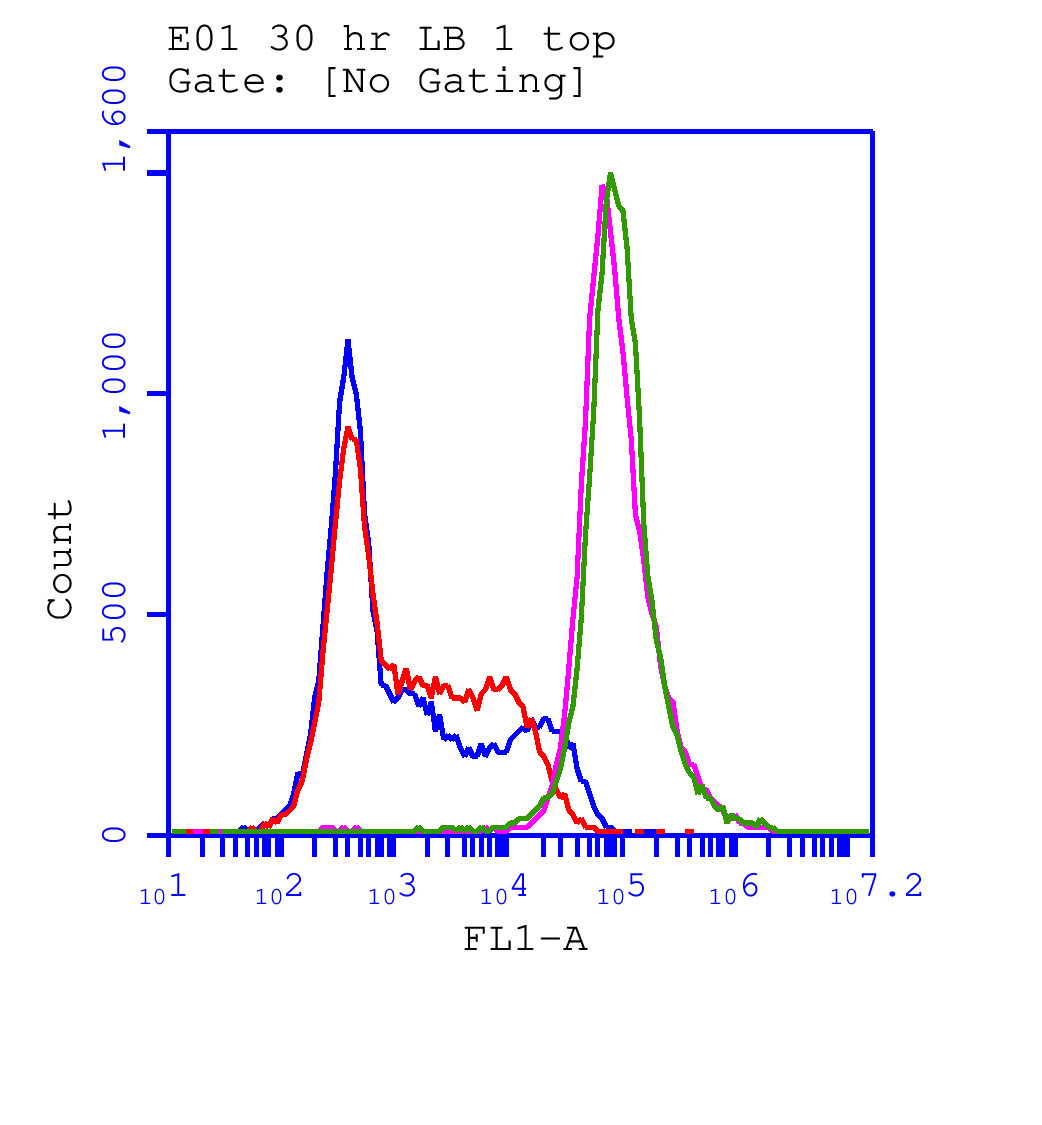

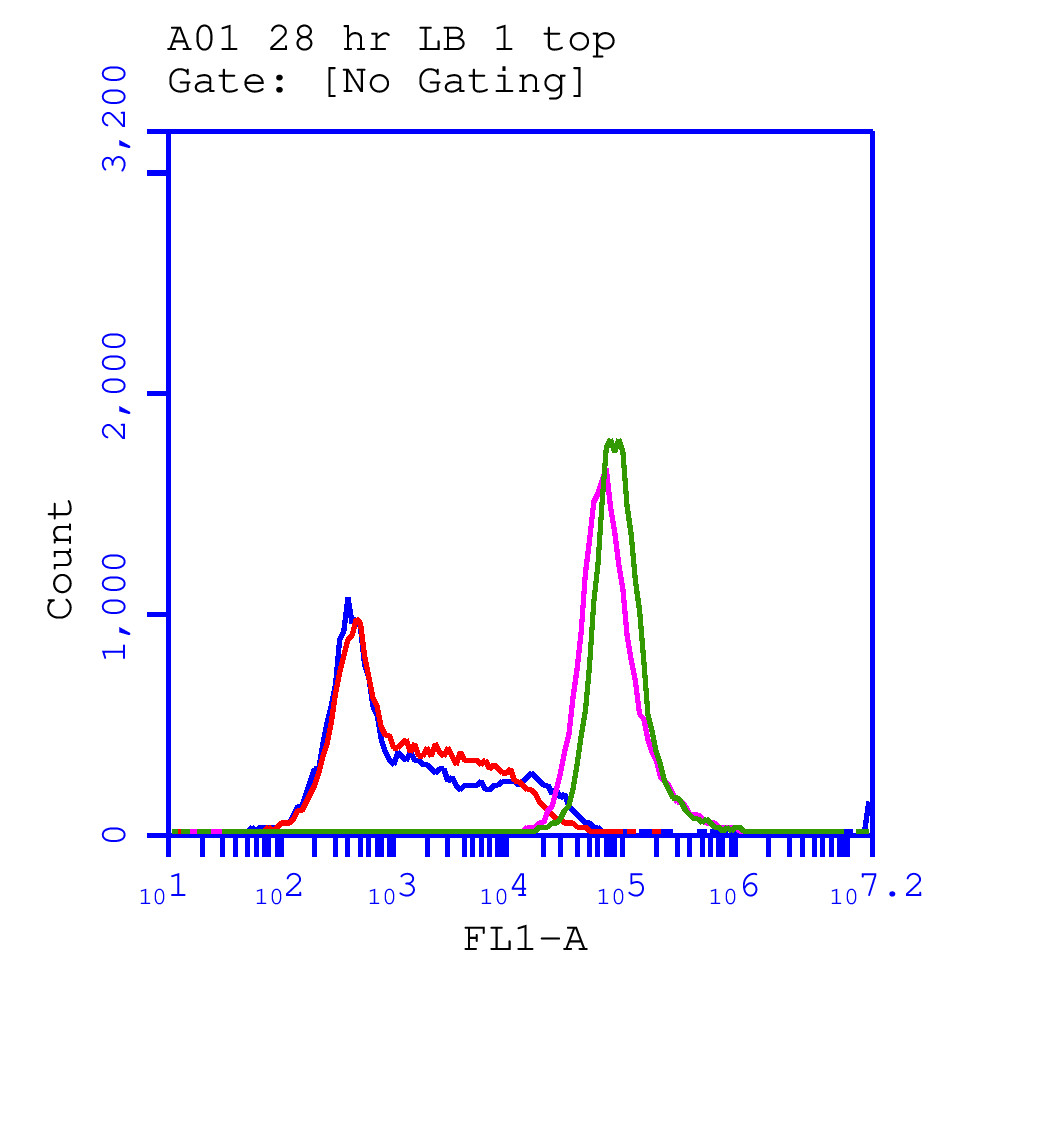

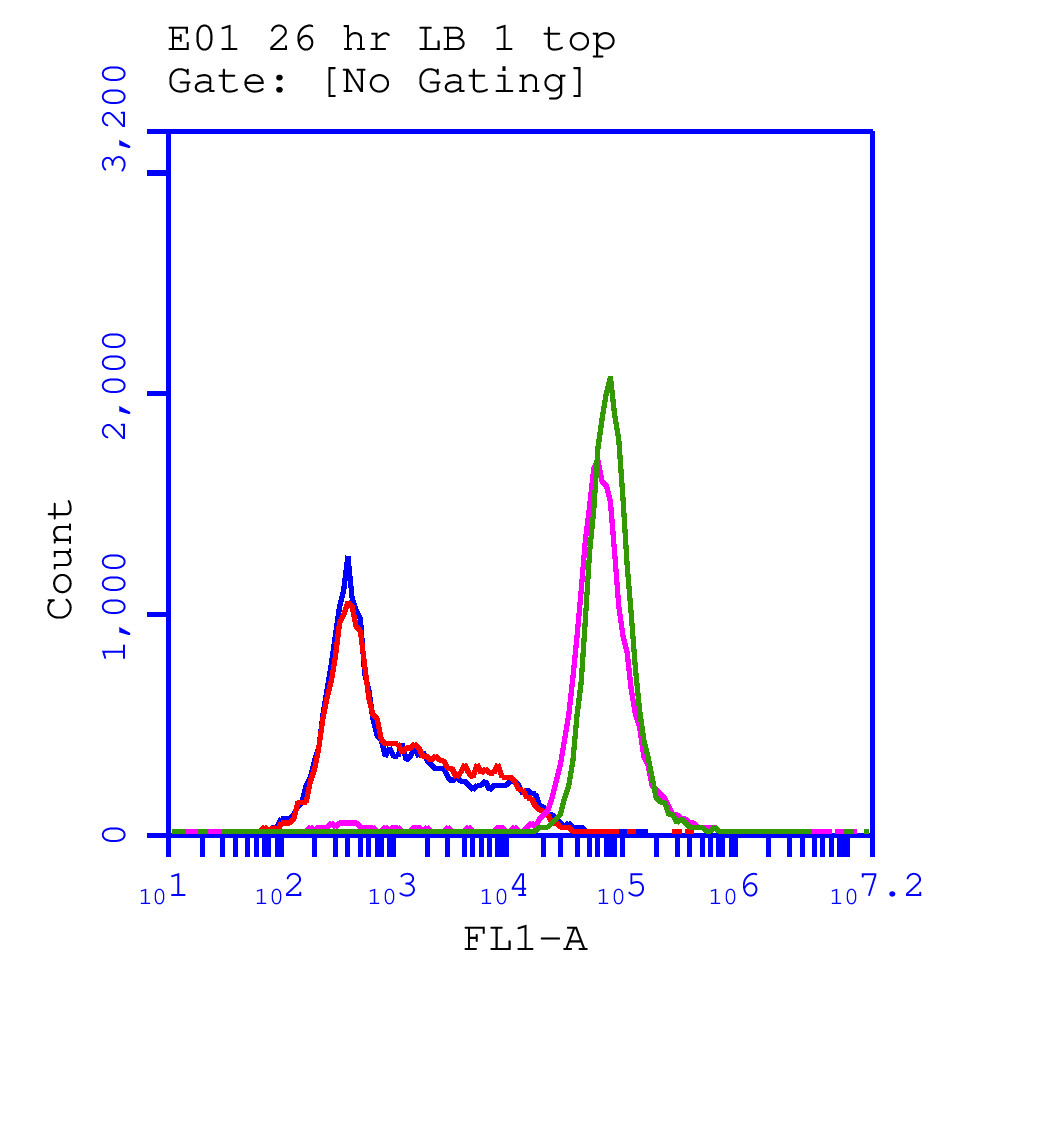

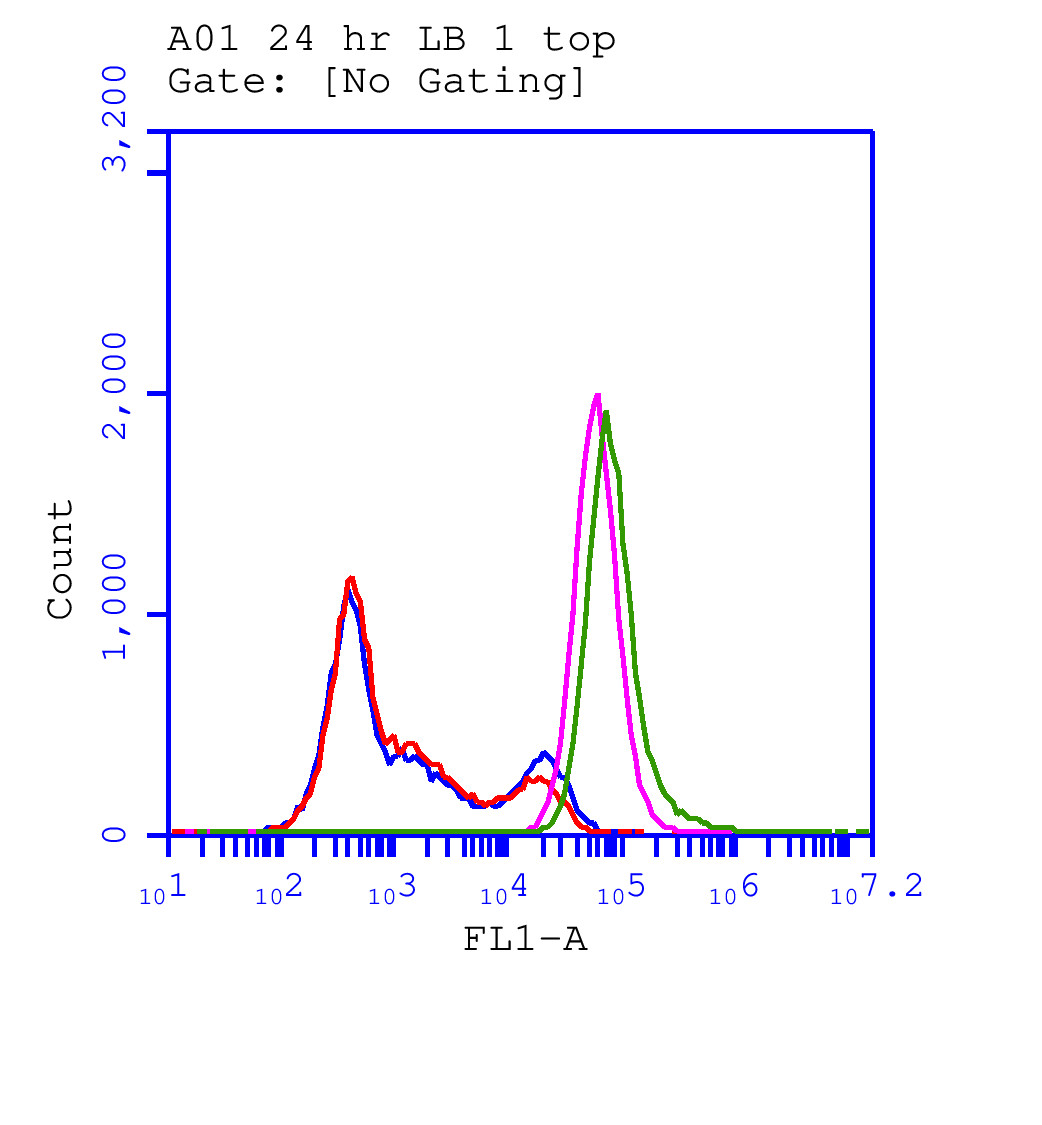

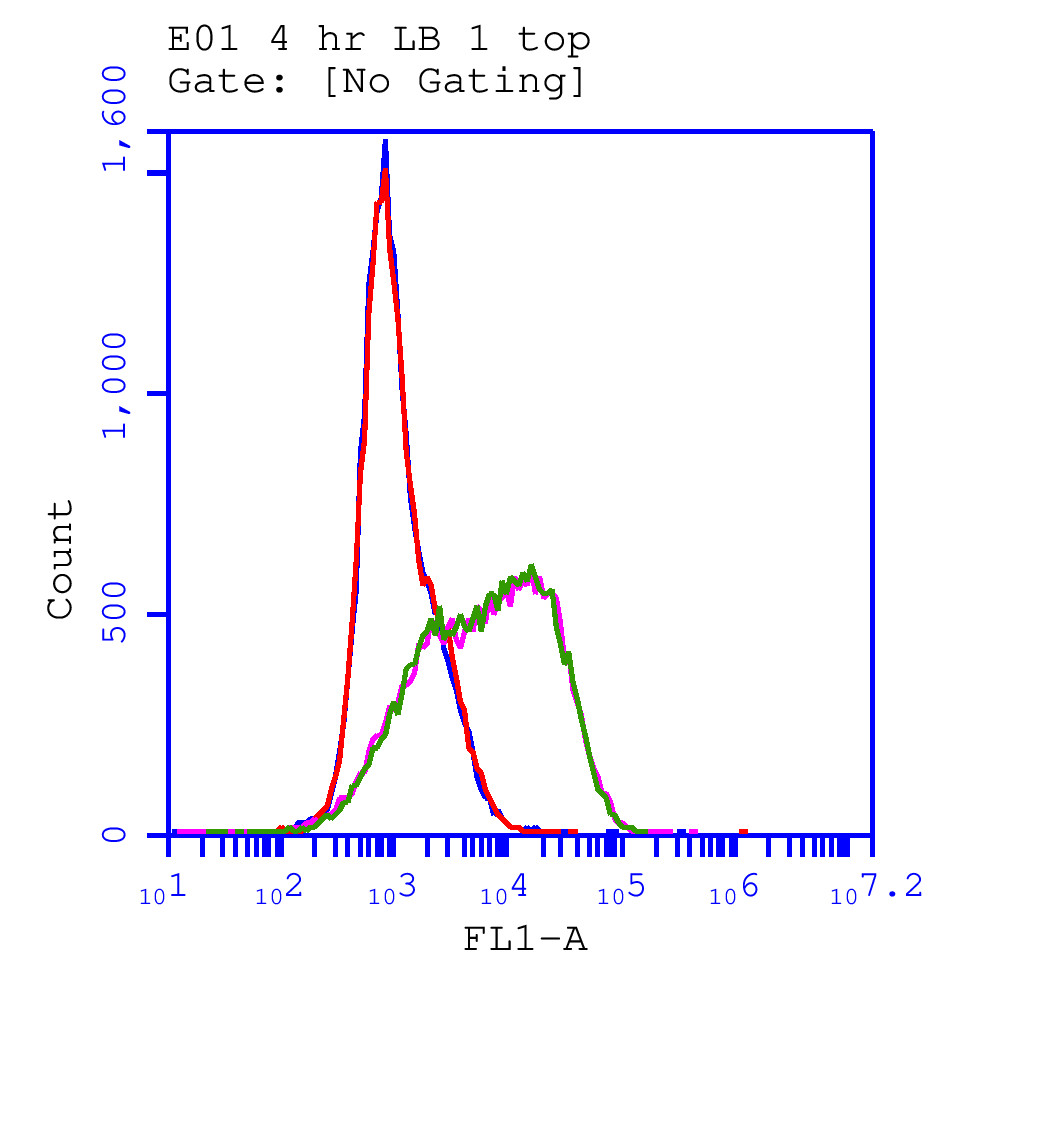

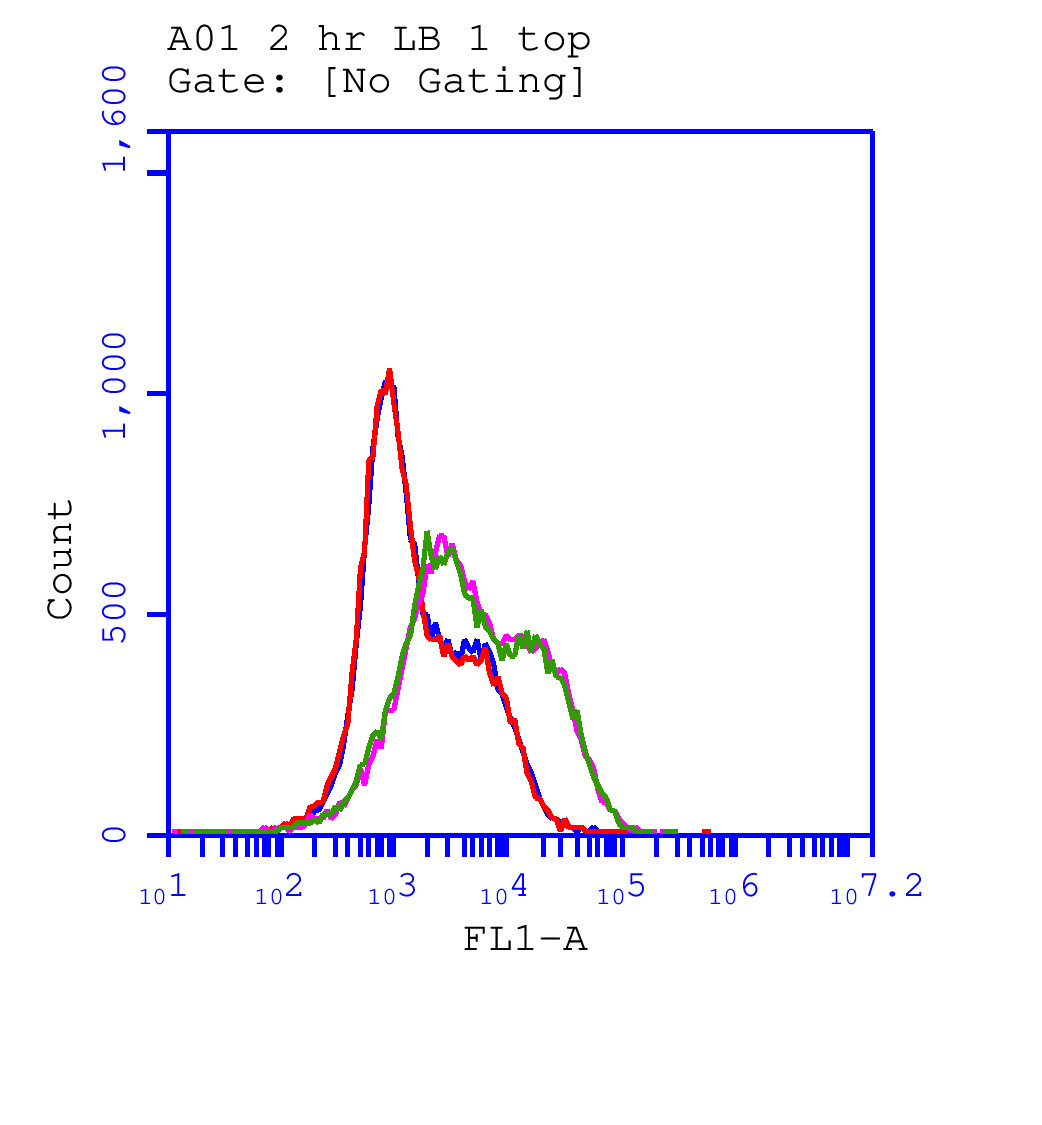


Top


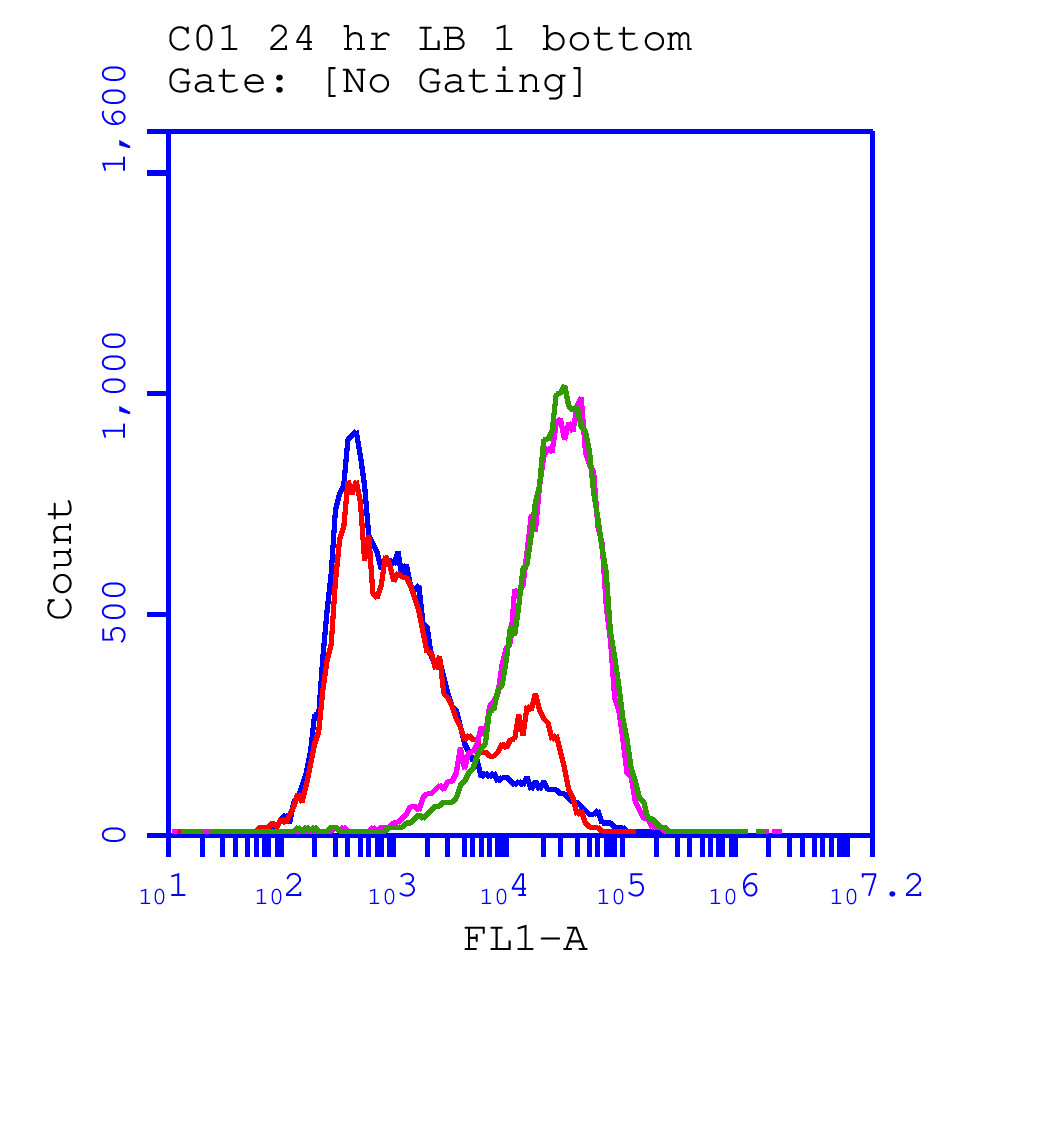

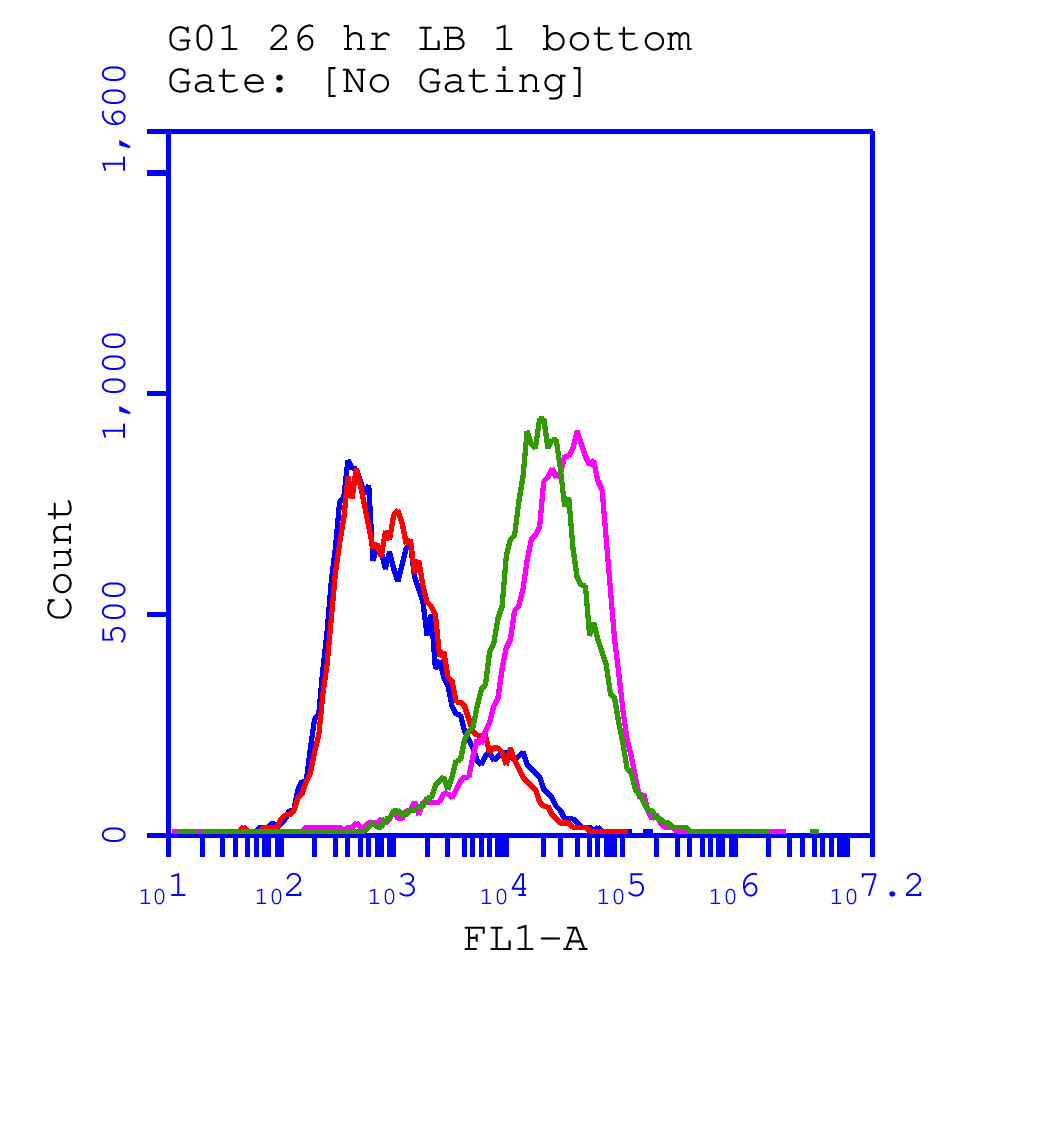

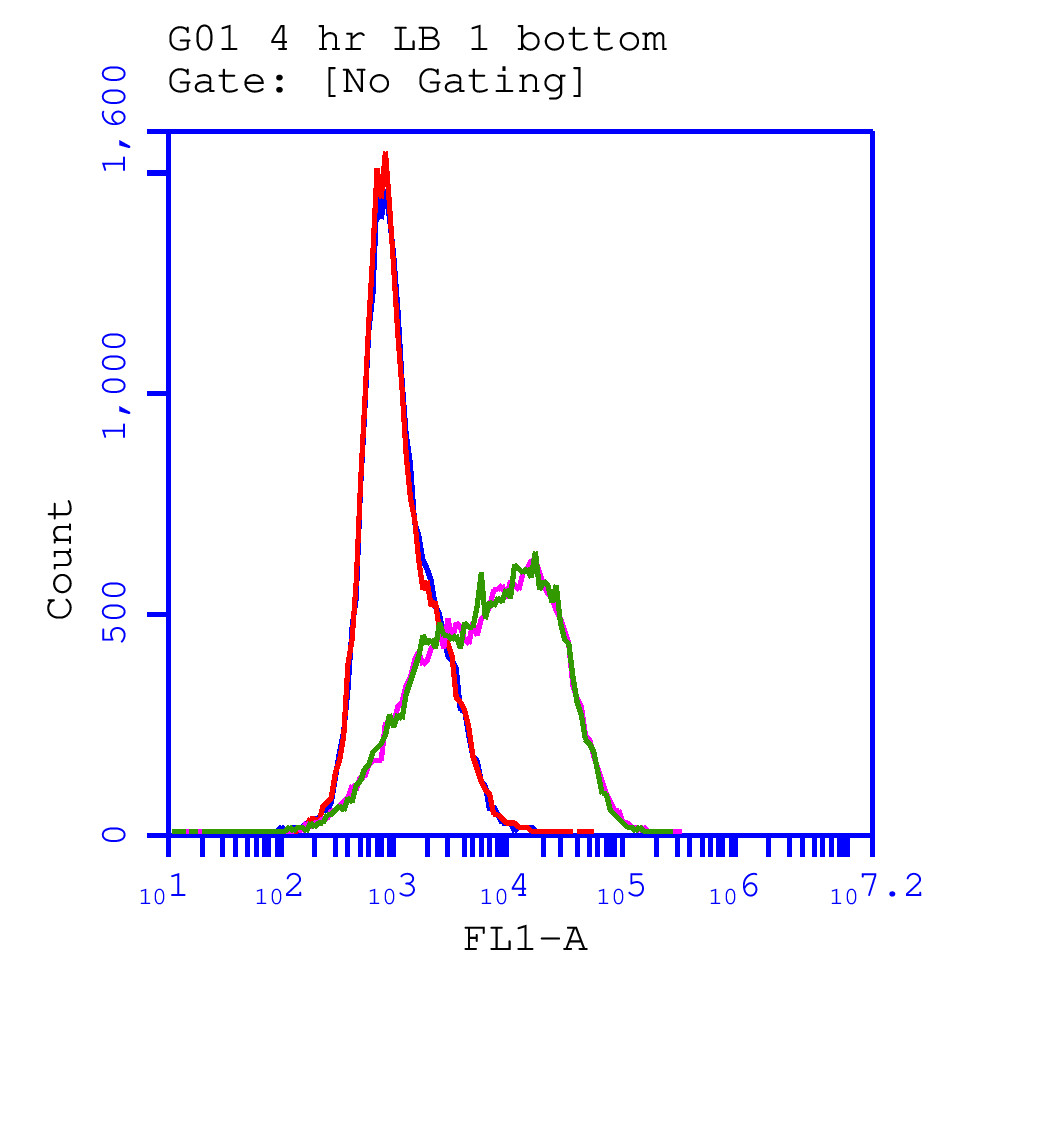

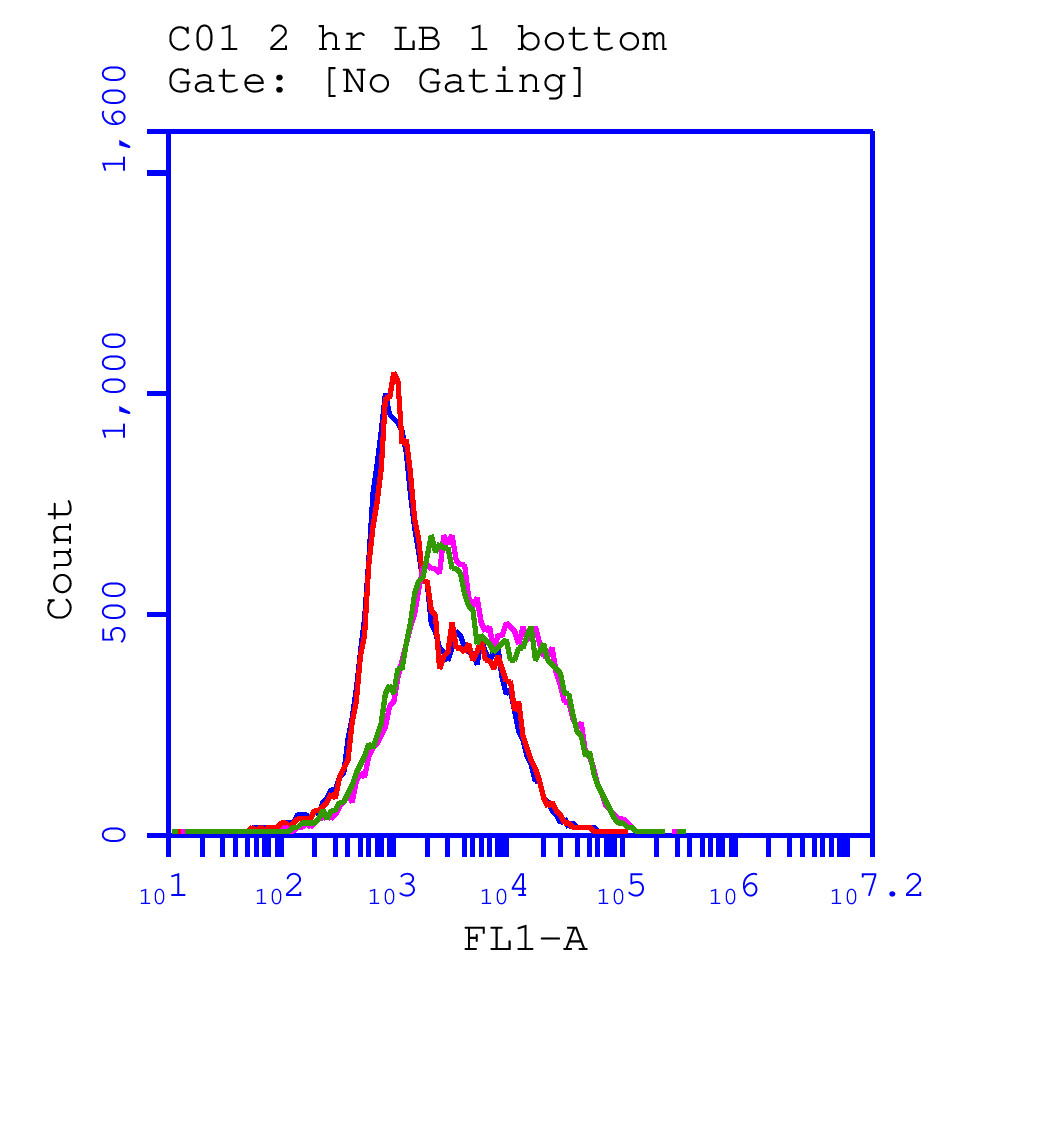

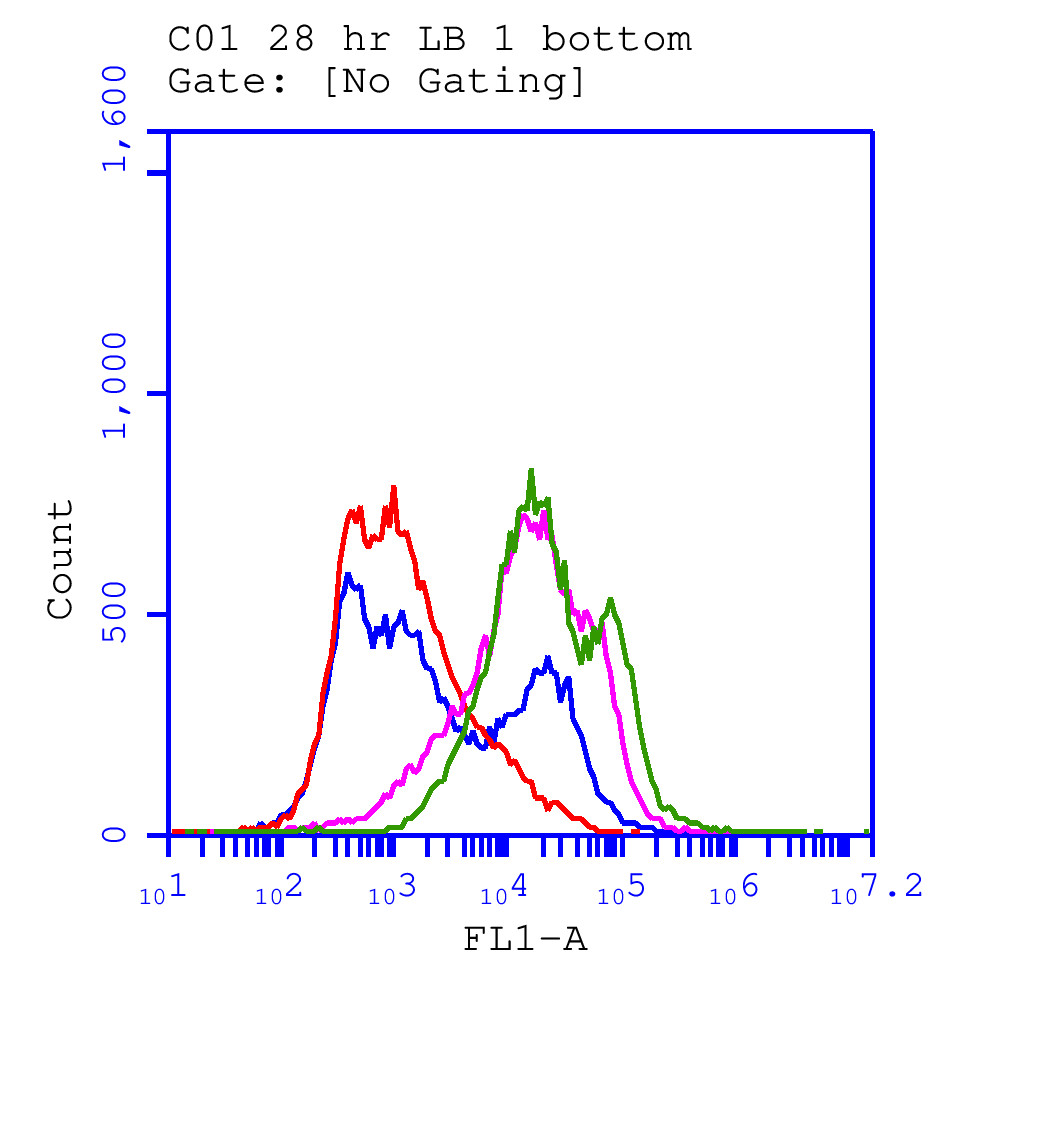

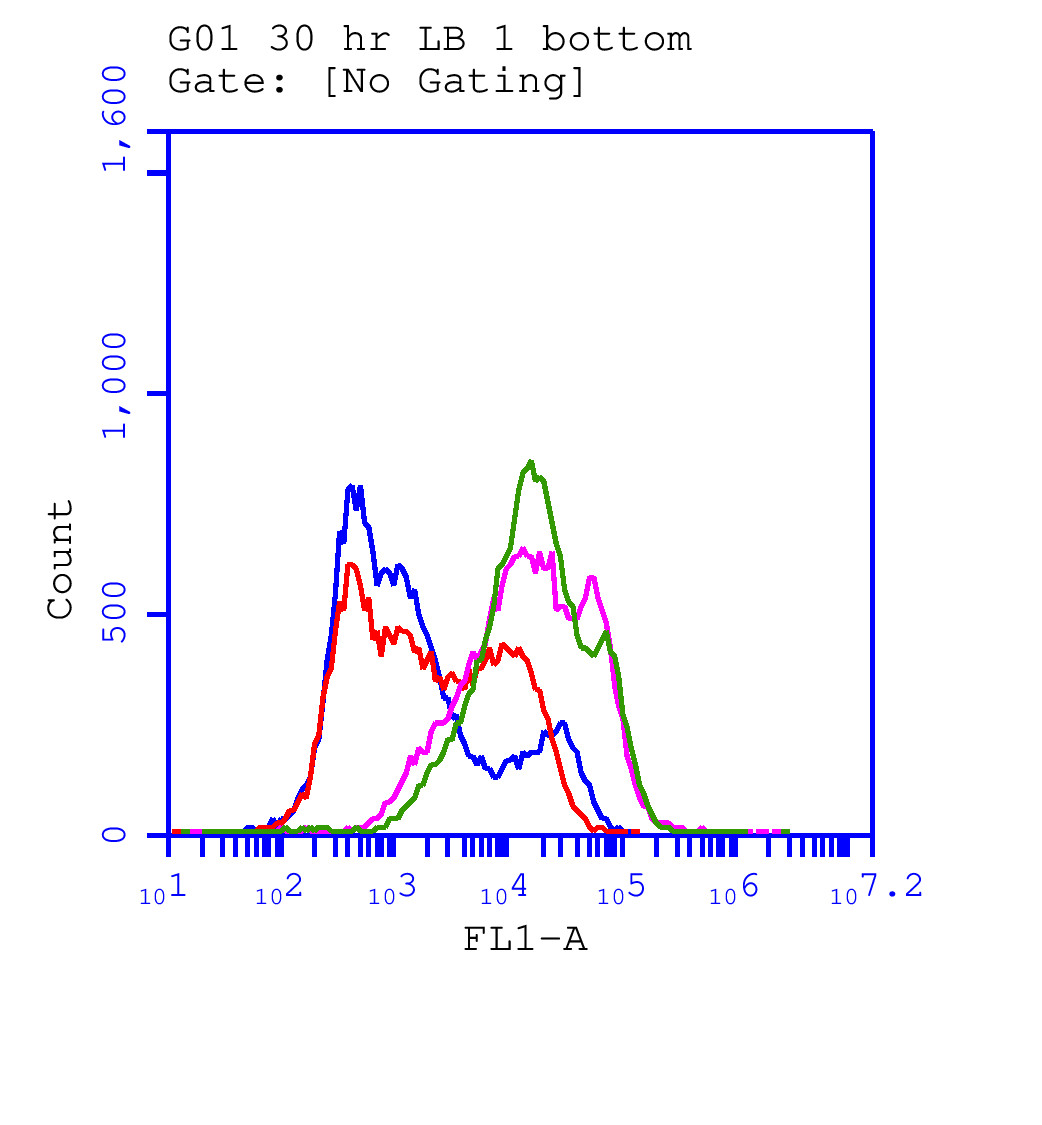


Bottom

LB

M63+

c)

56h

48h

32h

24h

8h

1h

Top

Bottom

0 mM

1 mM

10 mM

100 mM

M63+


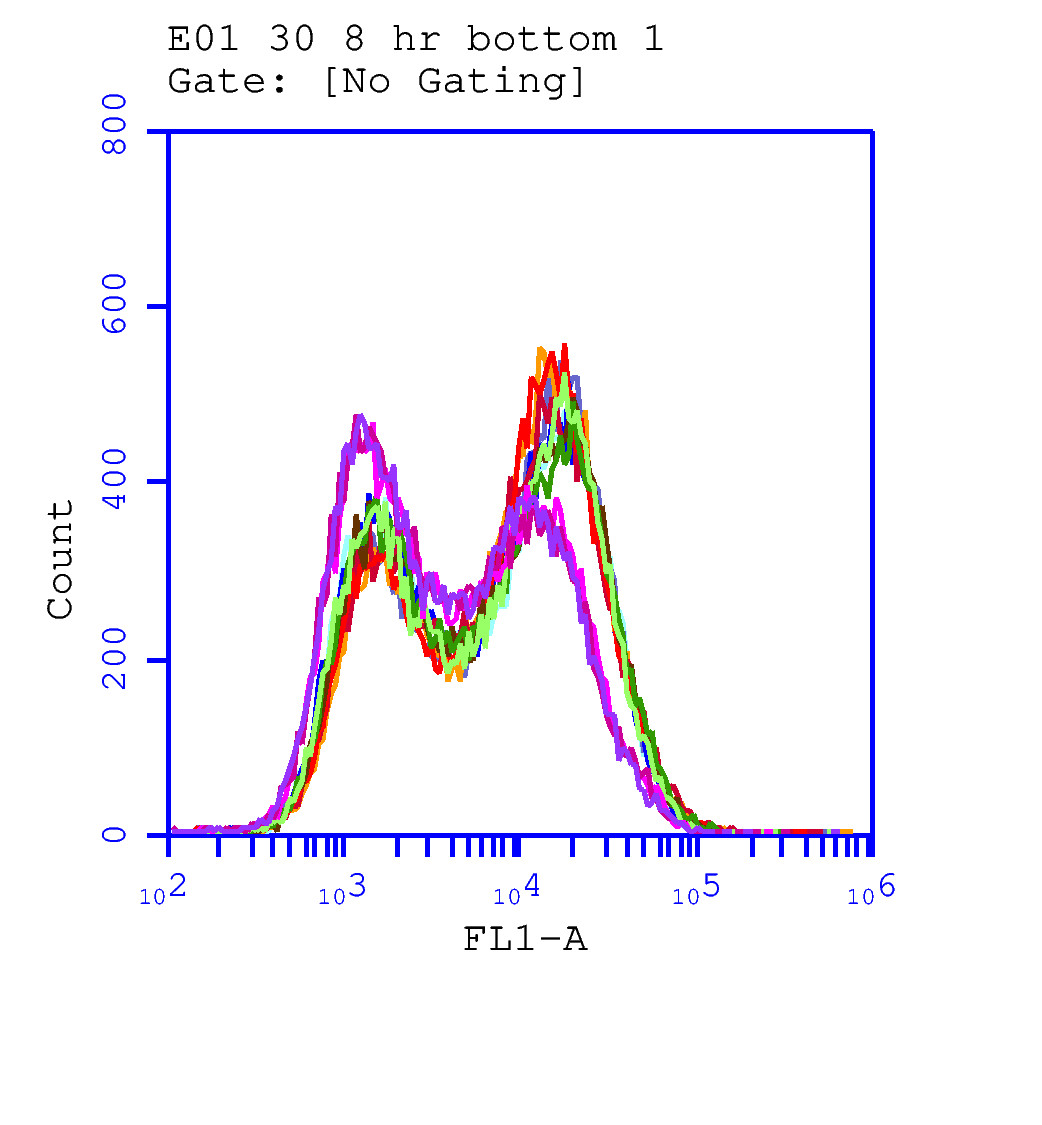

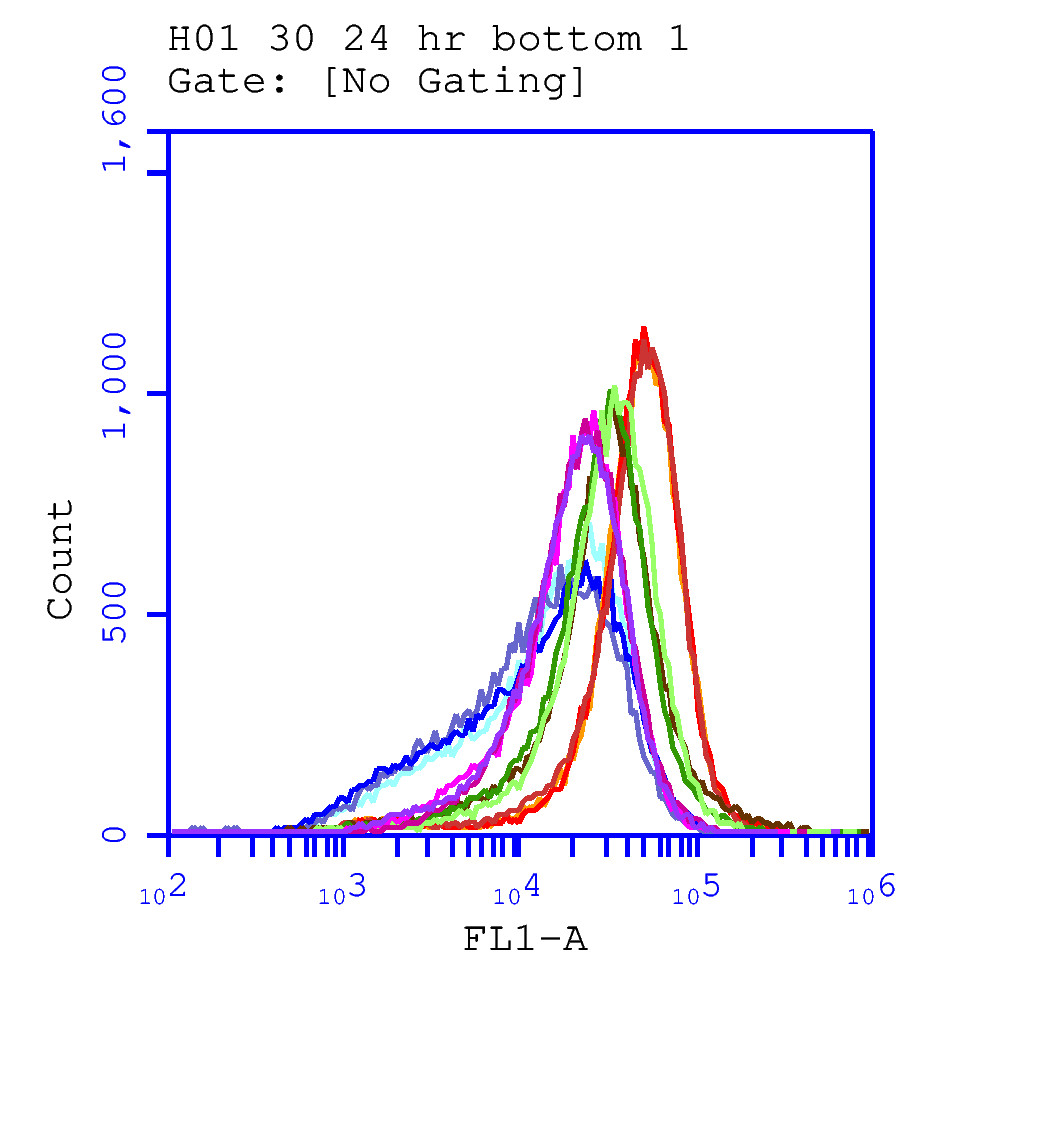

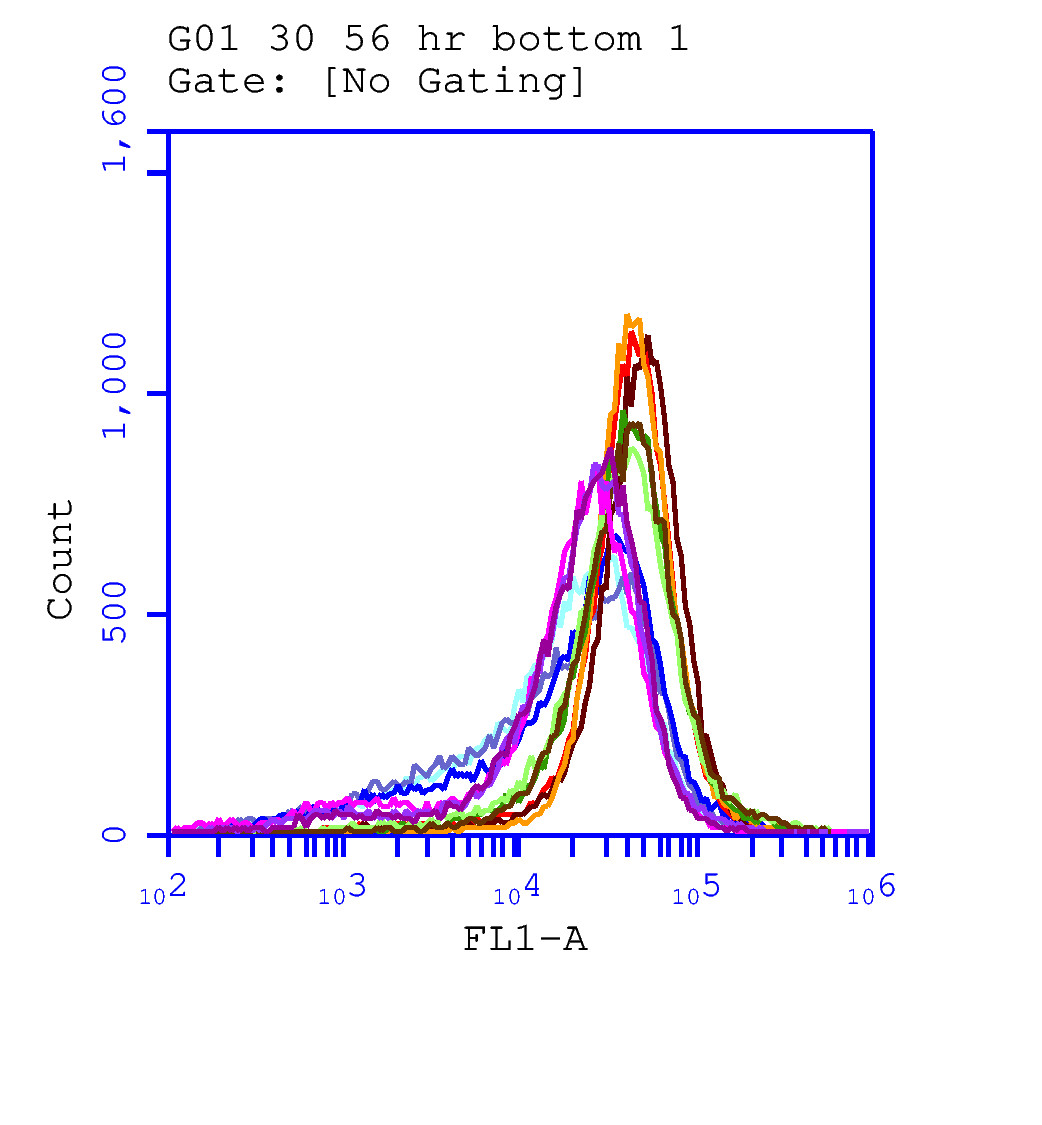

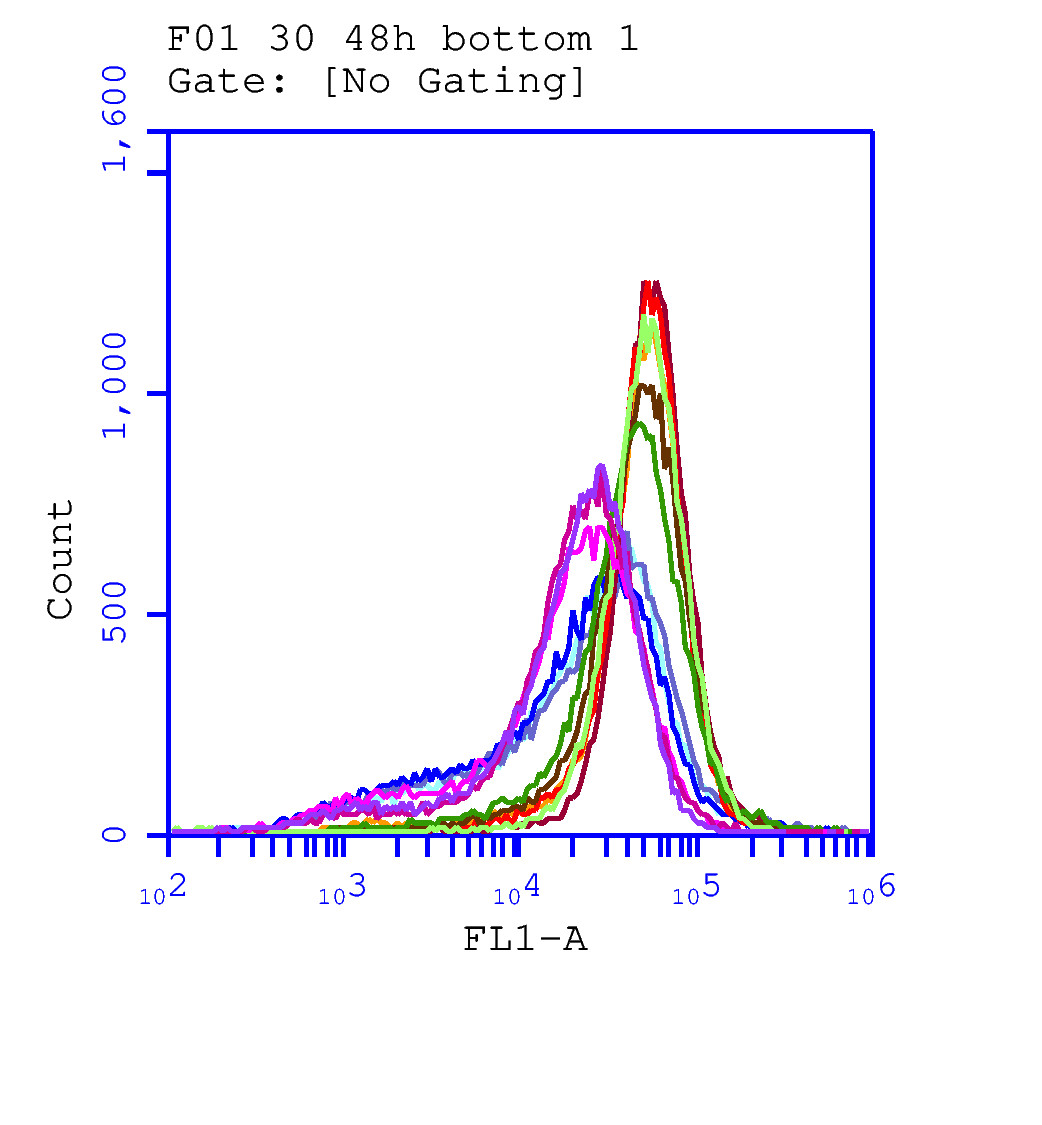

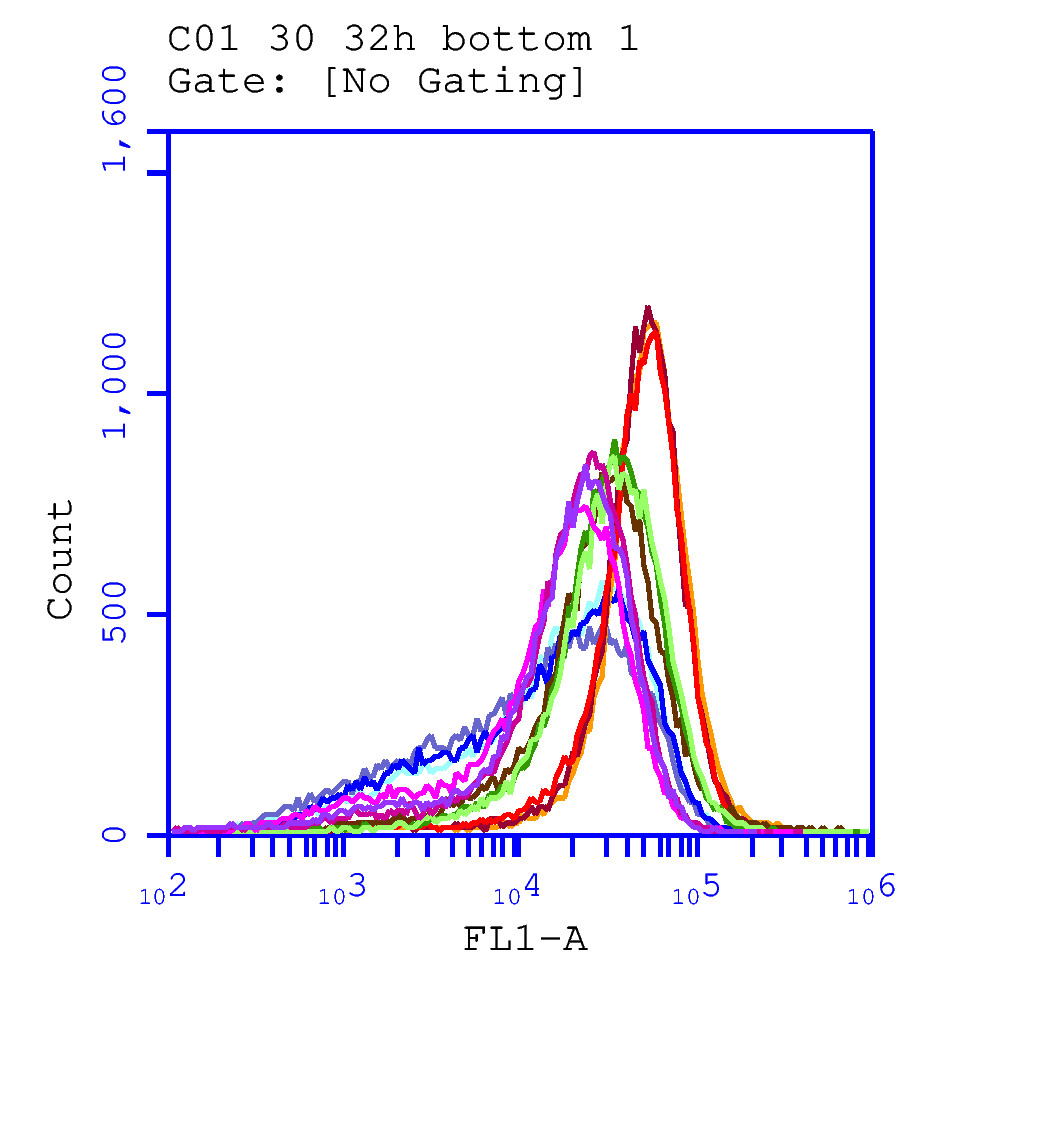

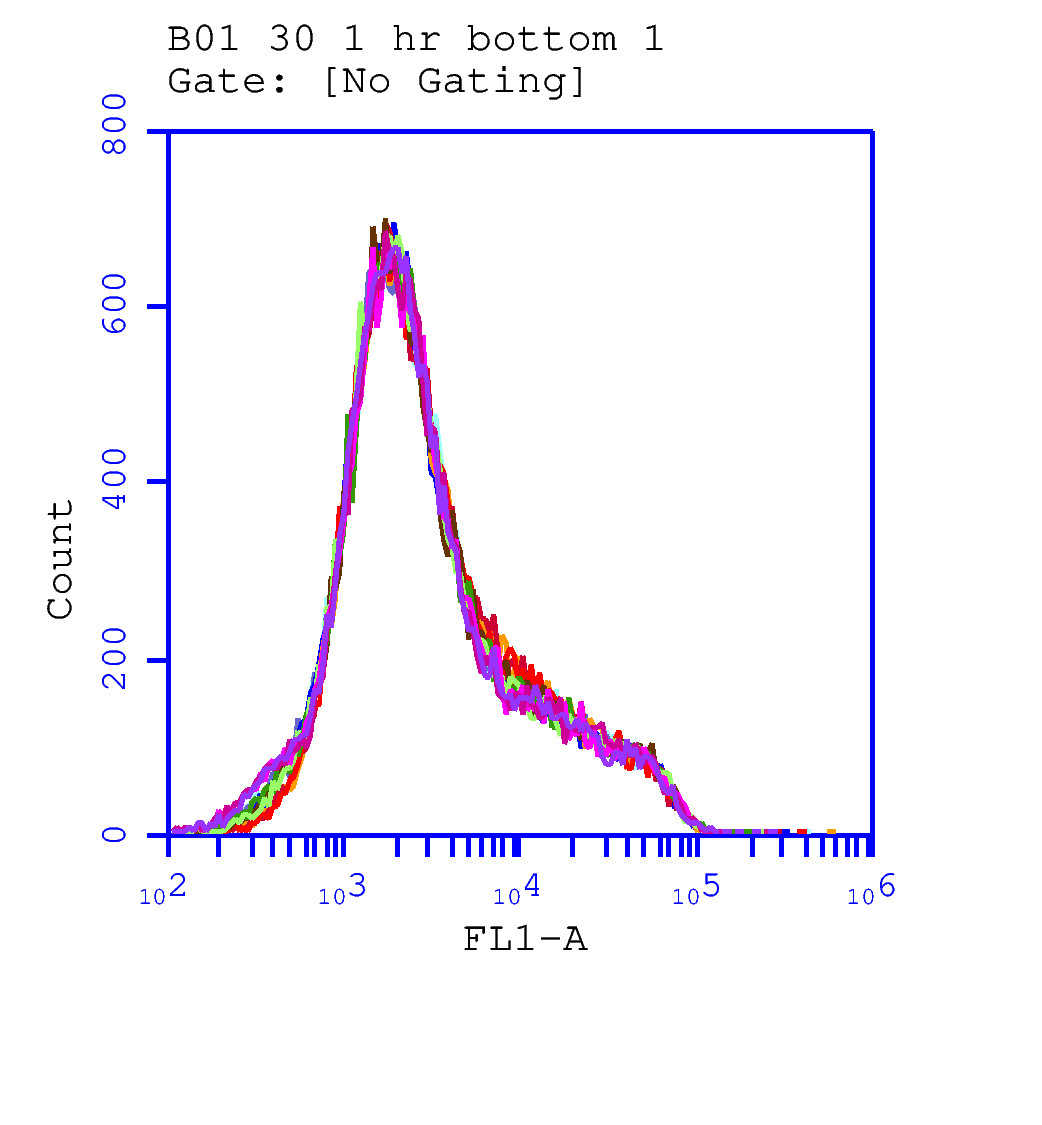

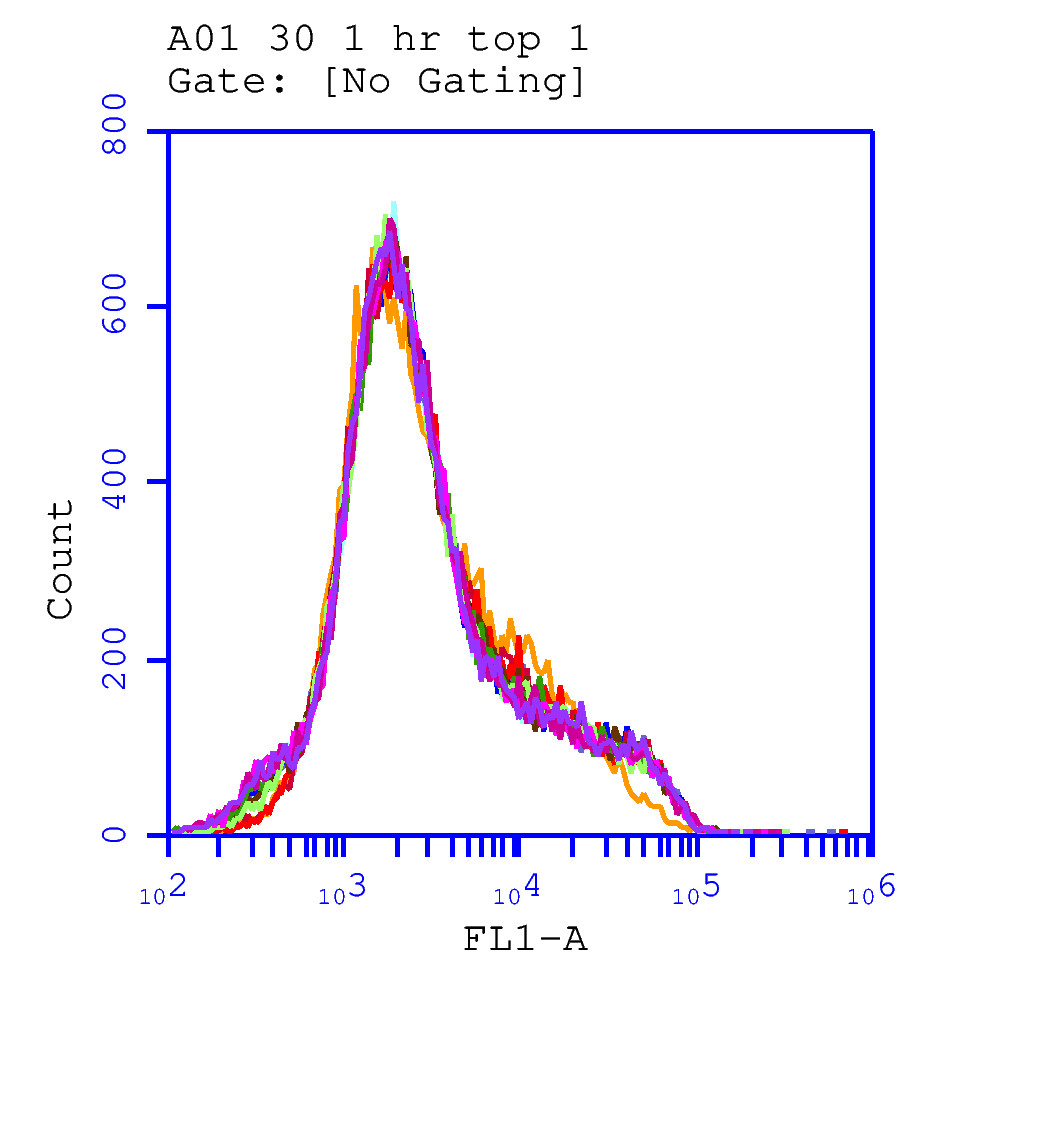

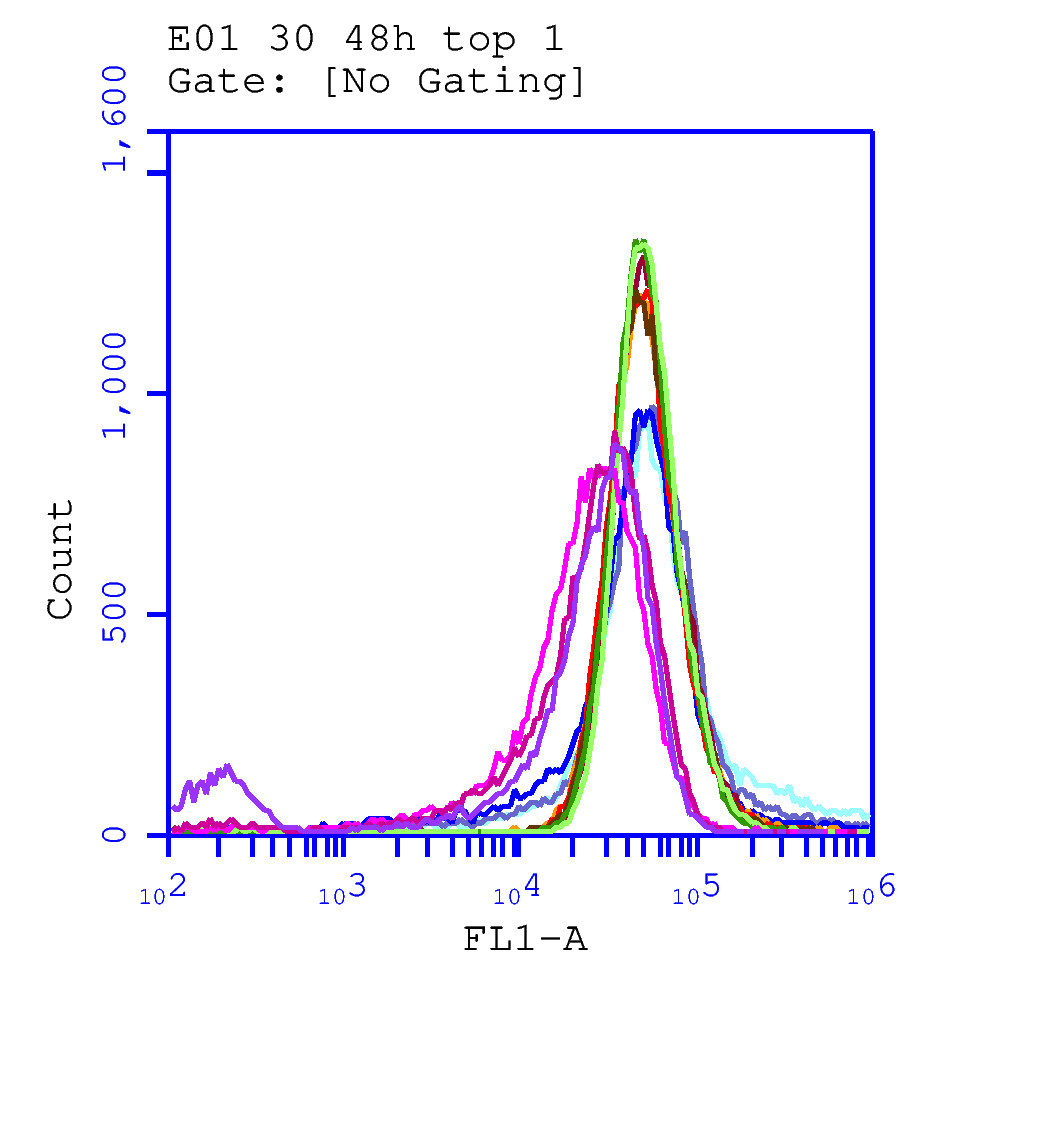

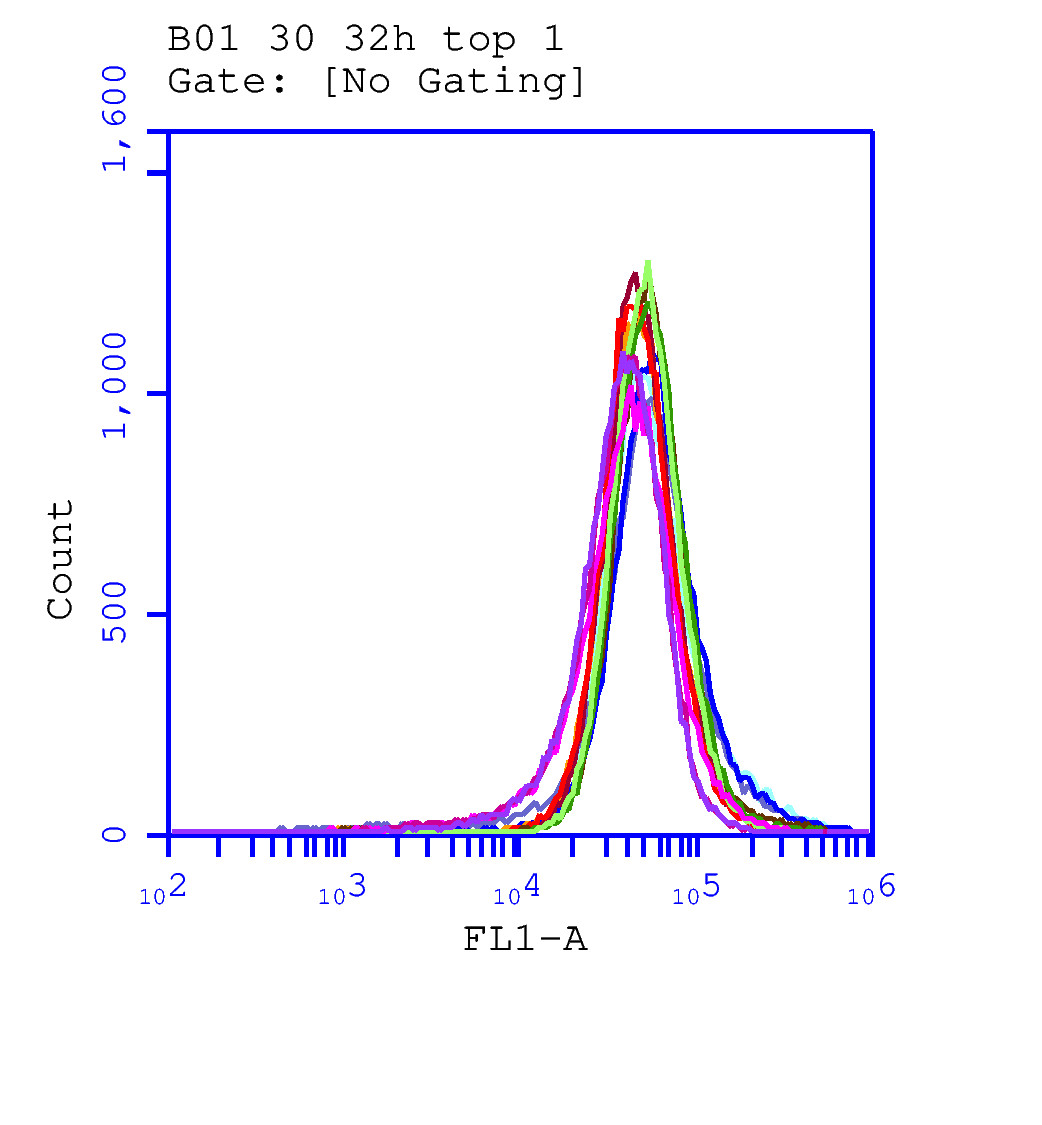

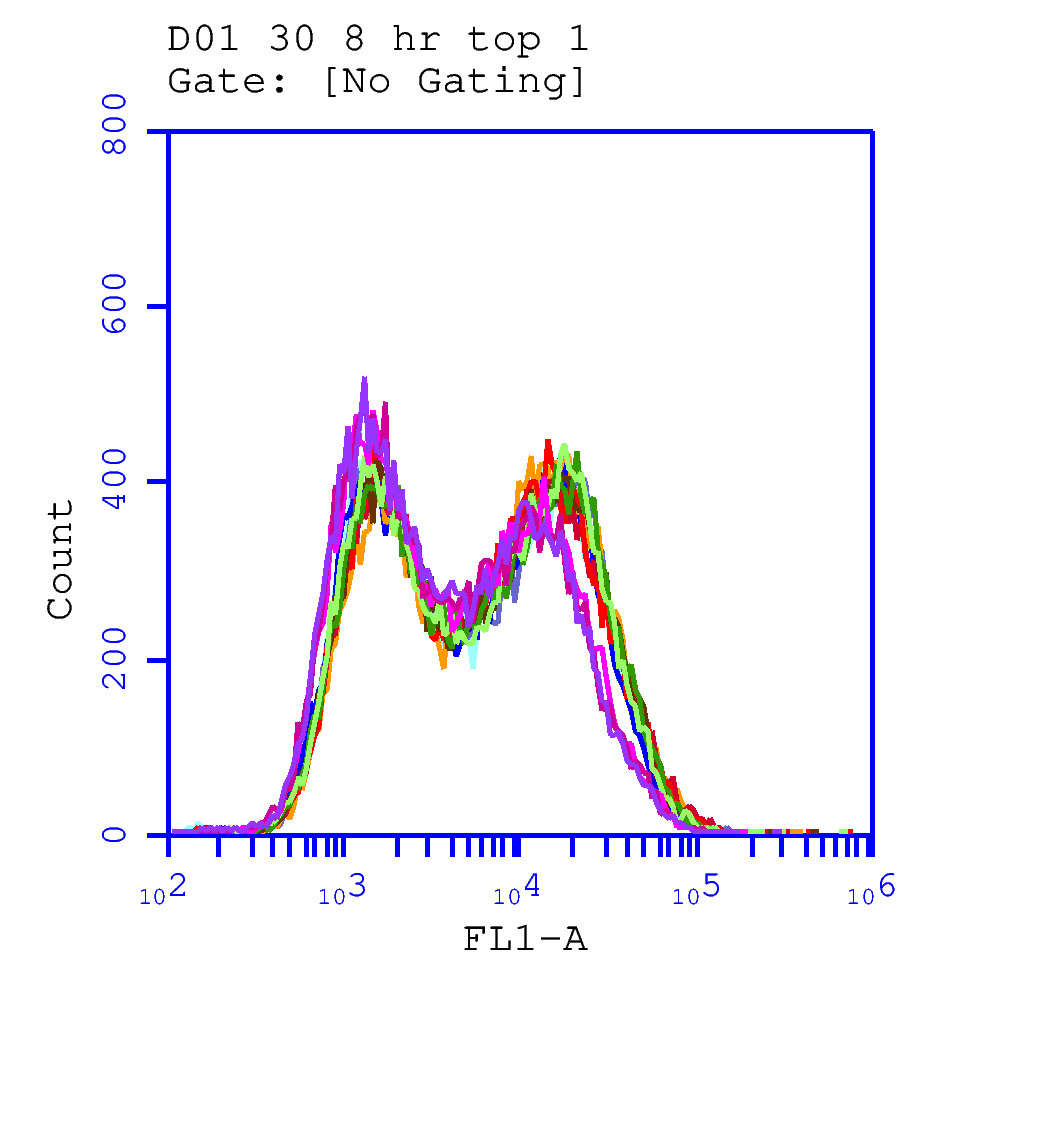

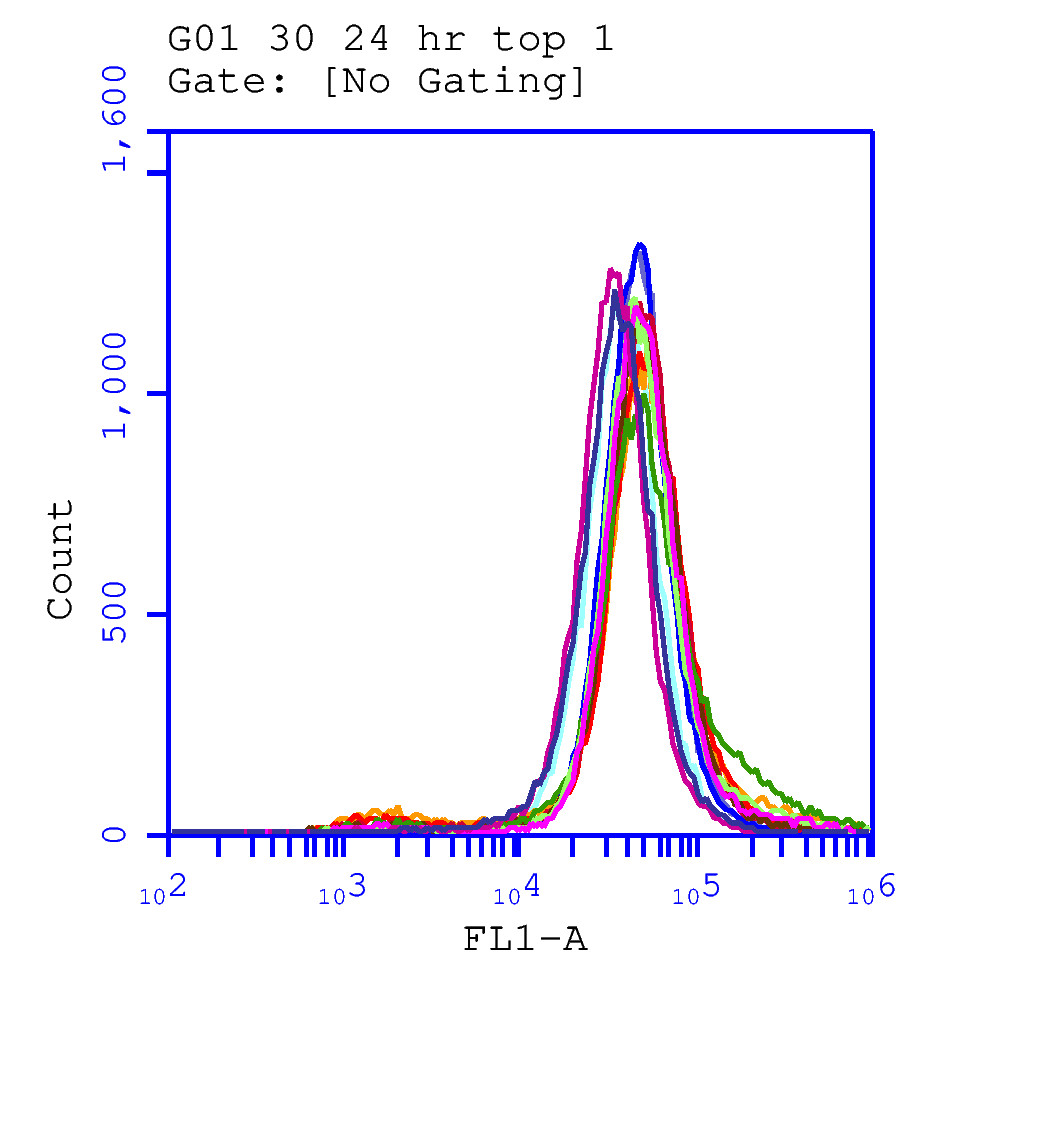

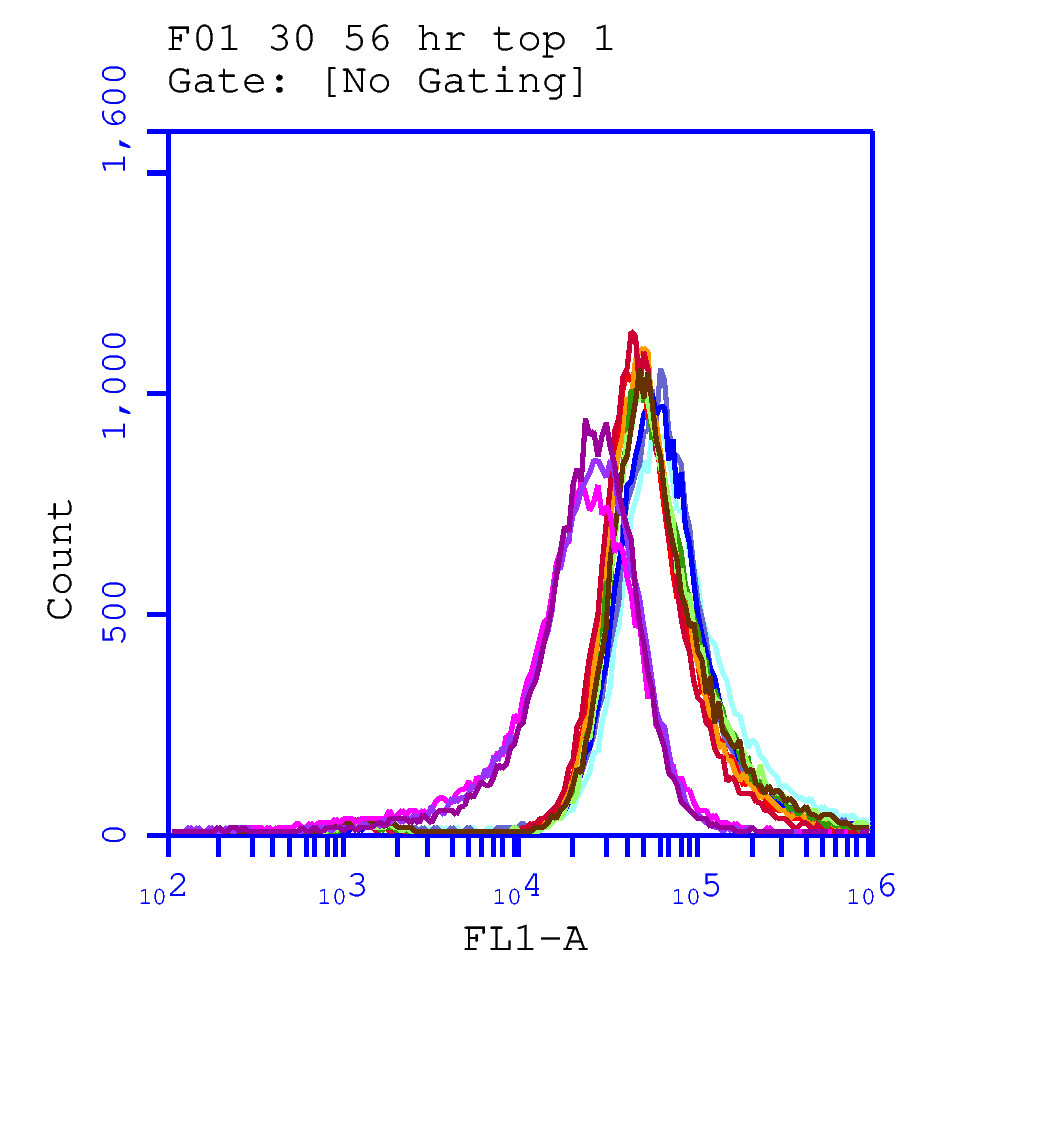


d)

Bottom

Top

56h

48h

32h

24h

8h

1h


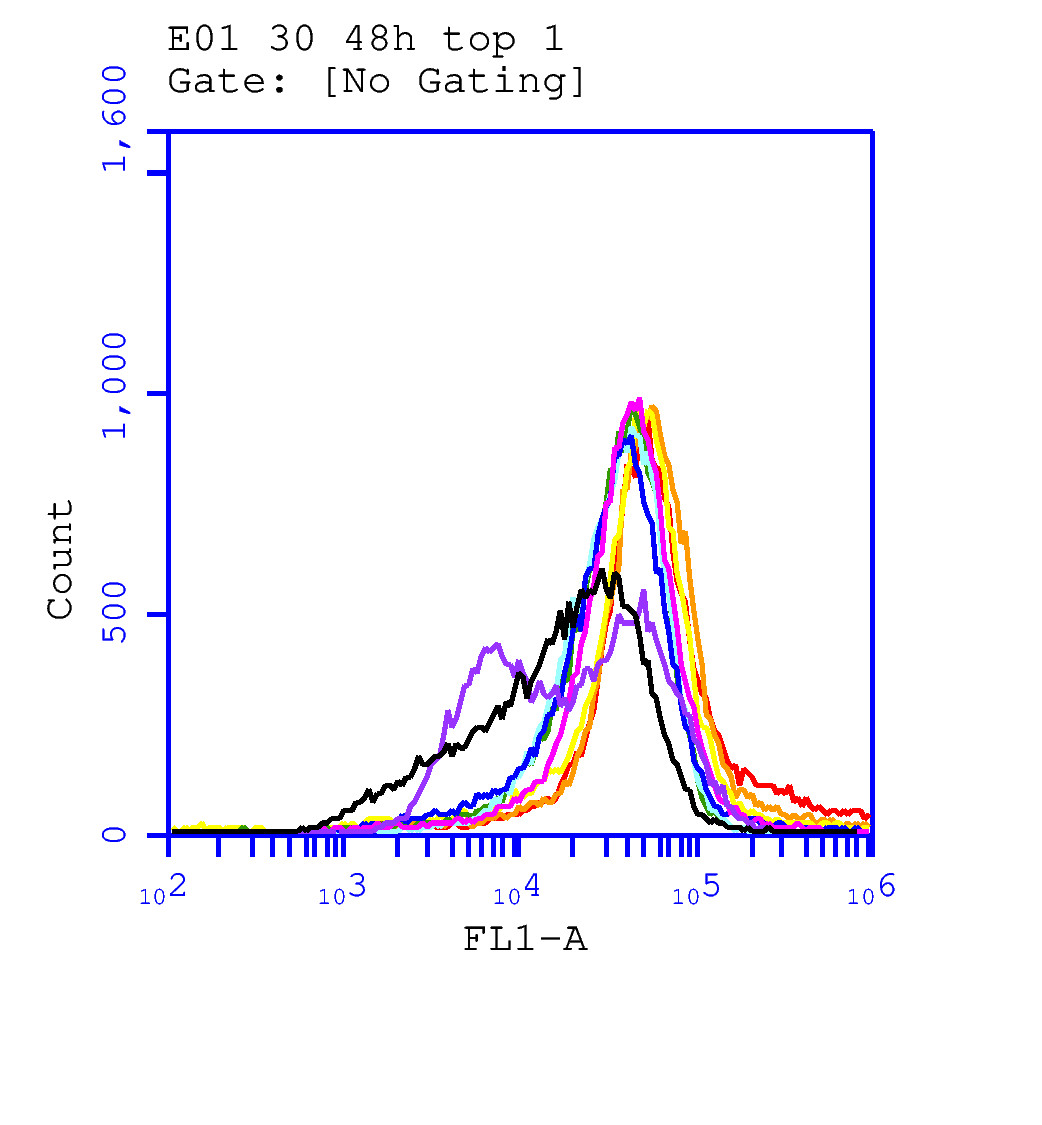

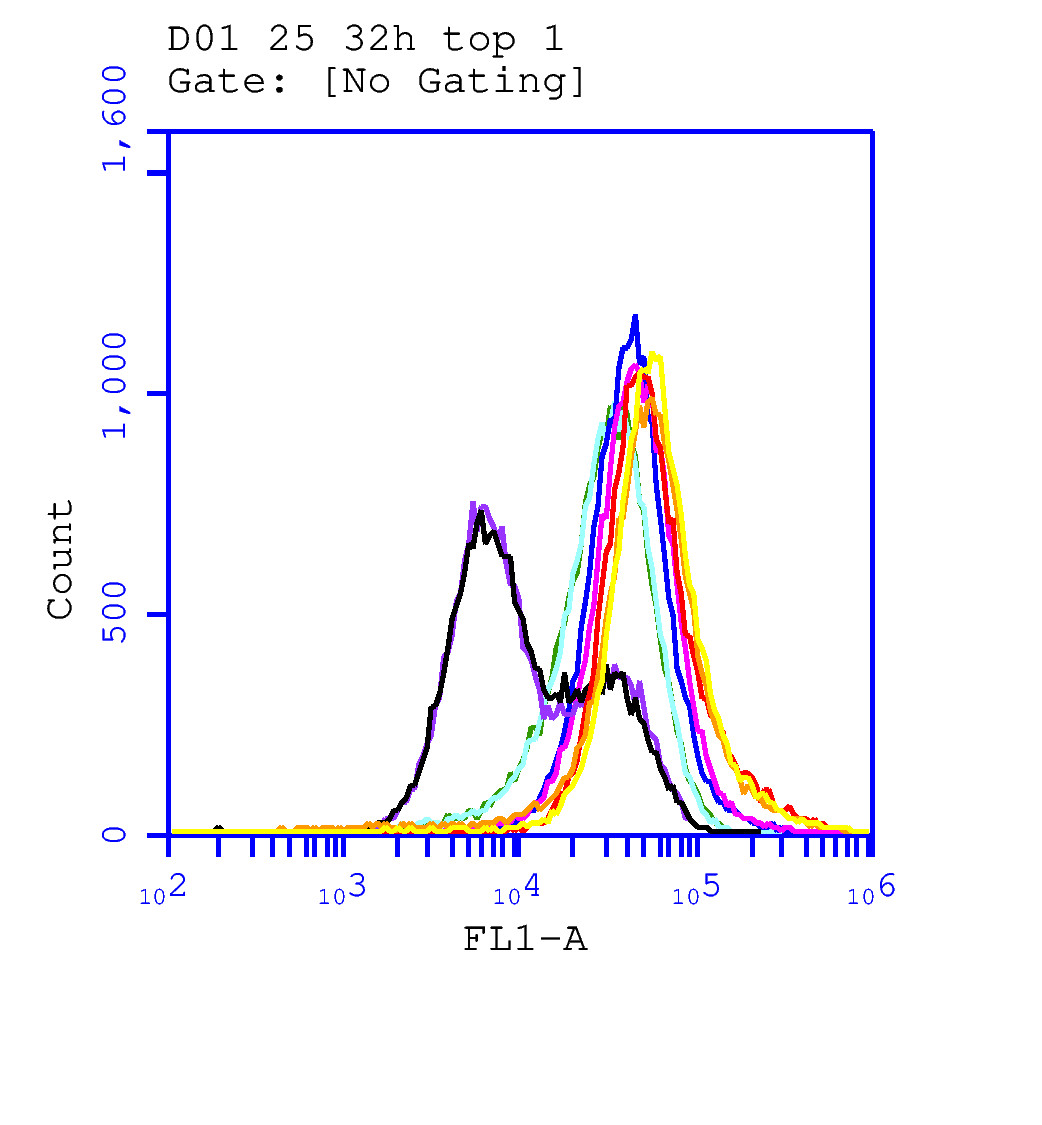

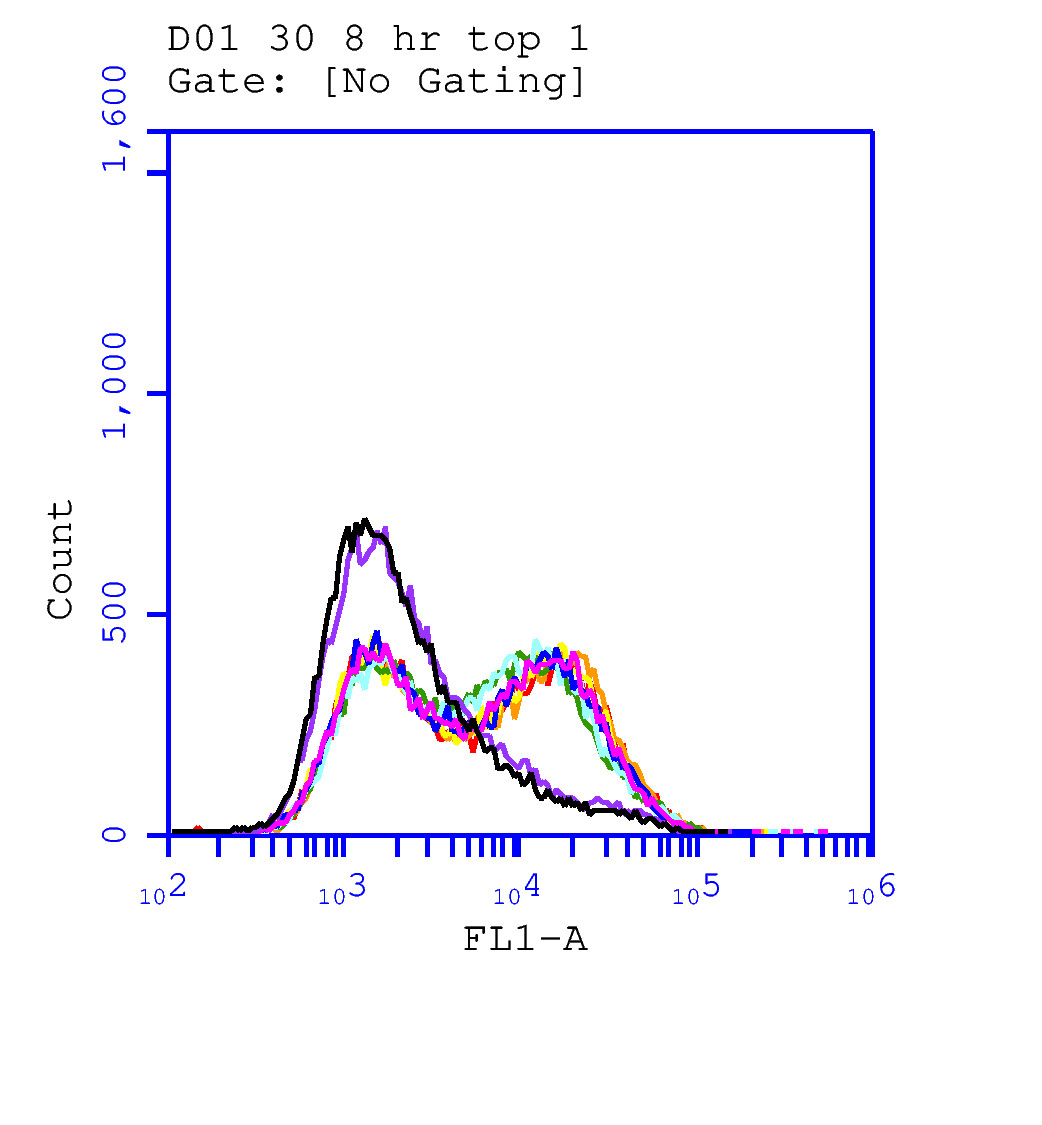

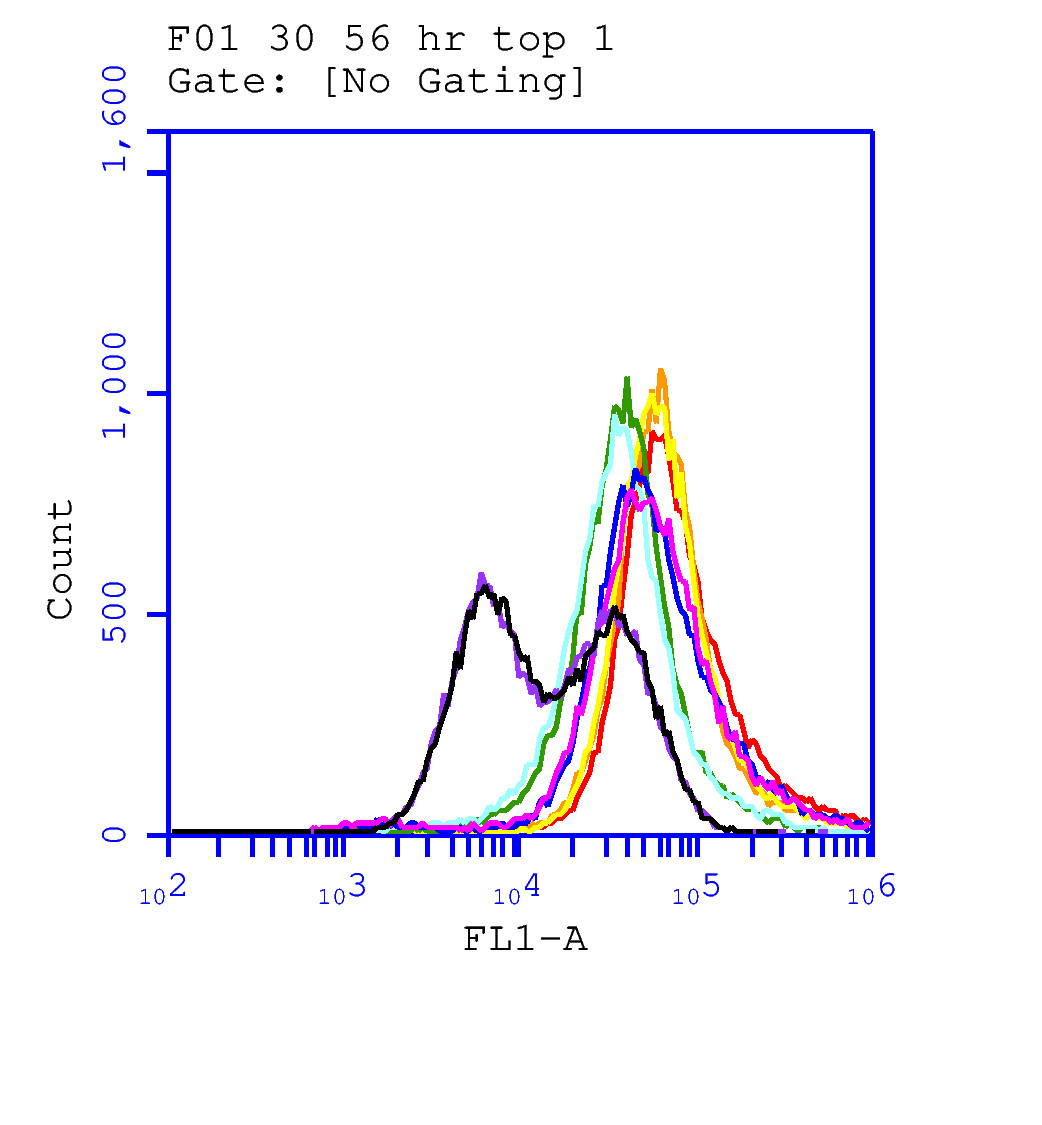

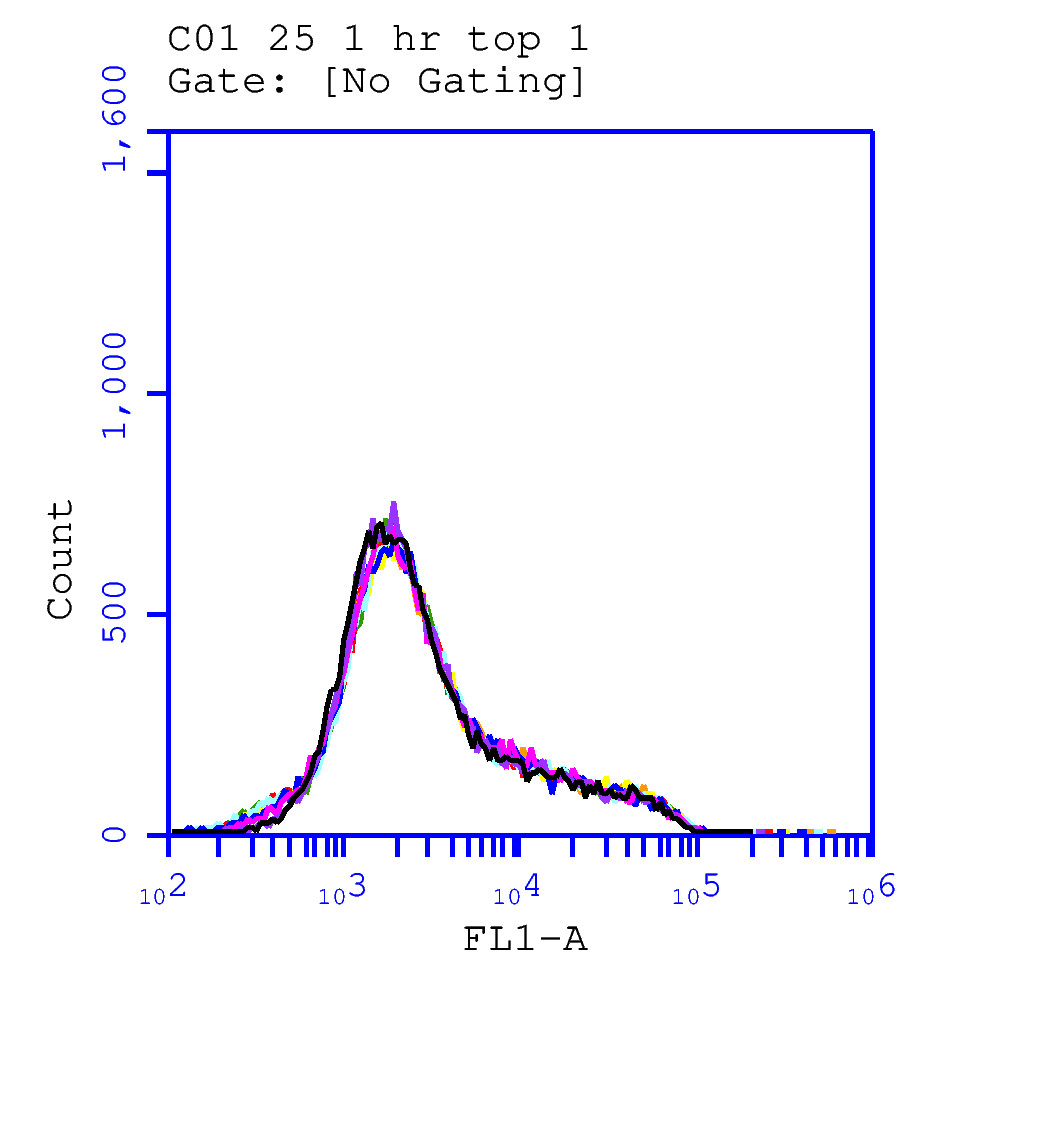

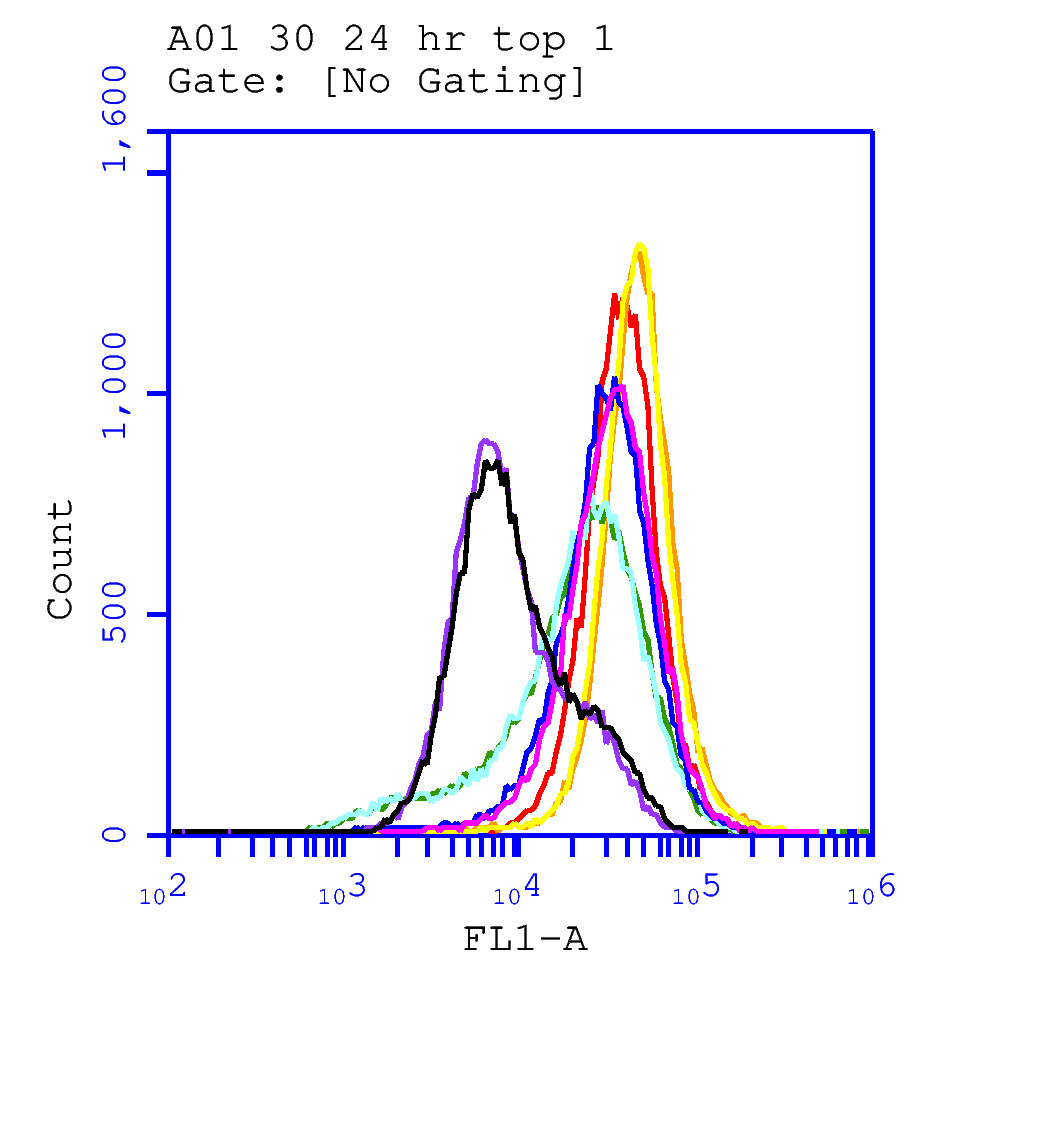


30 °C

25 °C

28 °C

37 °C

M63+


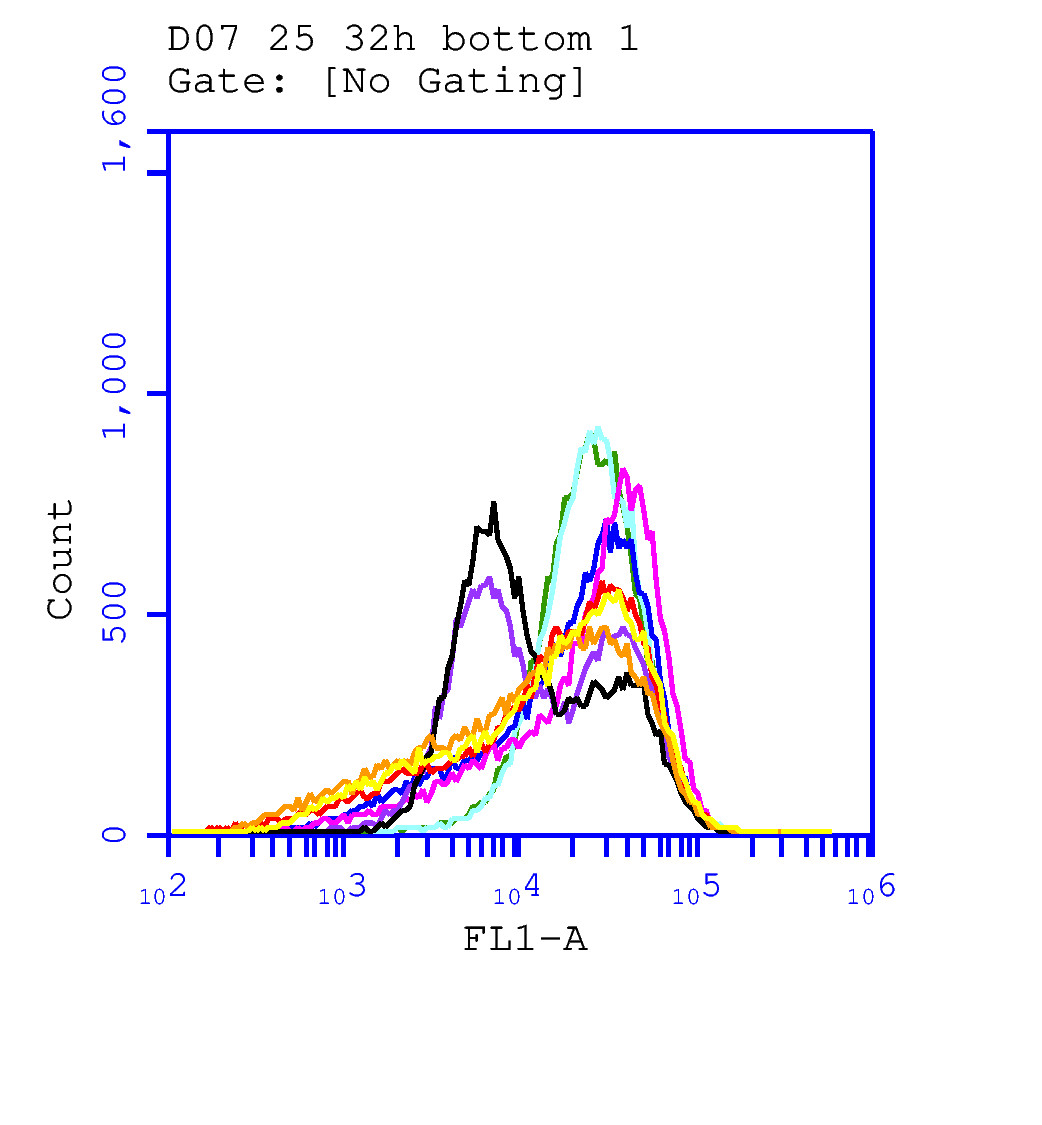

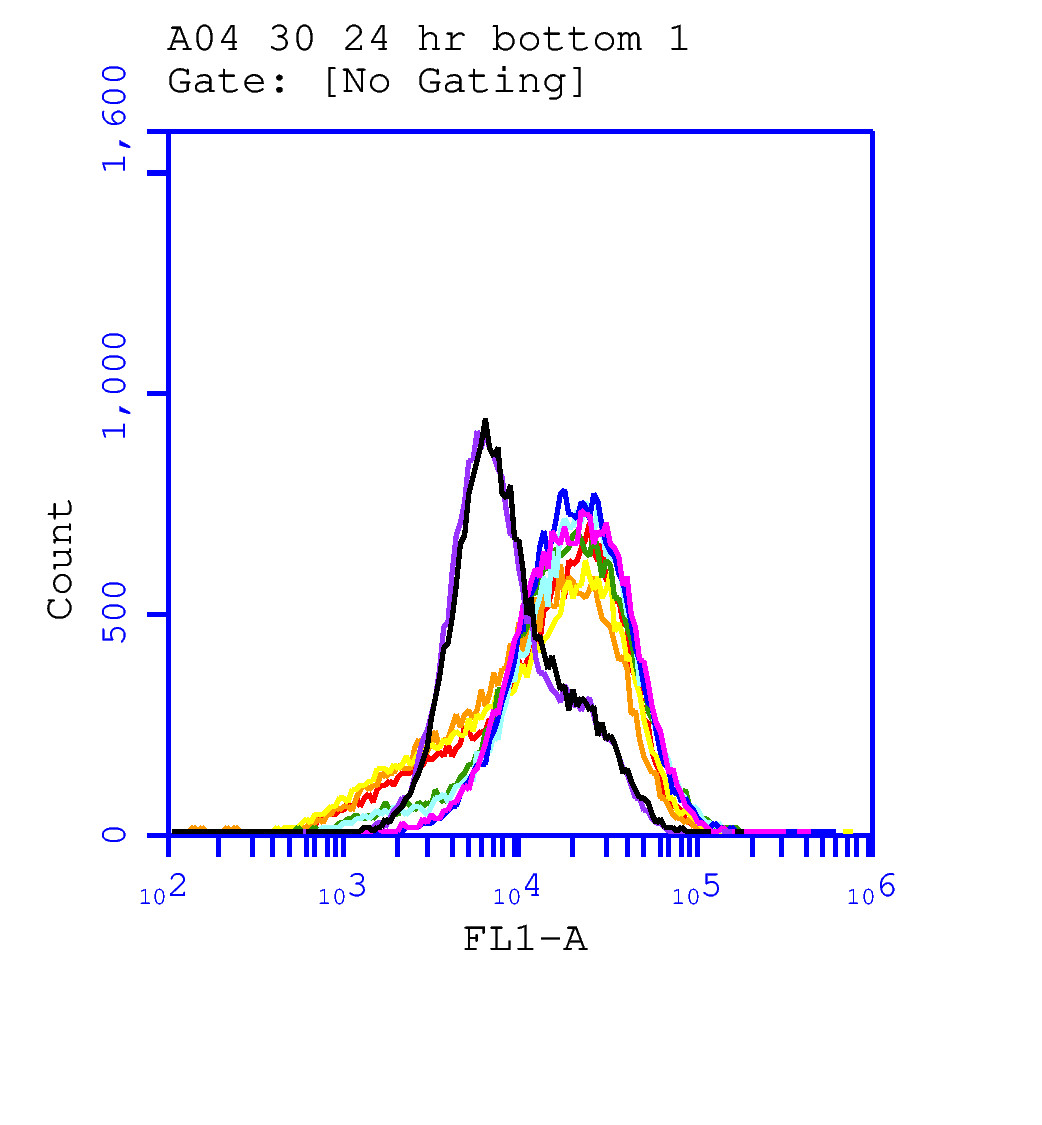

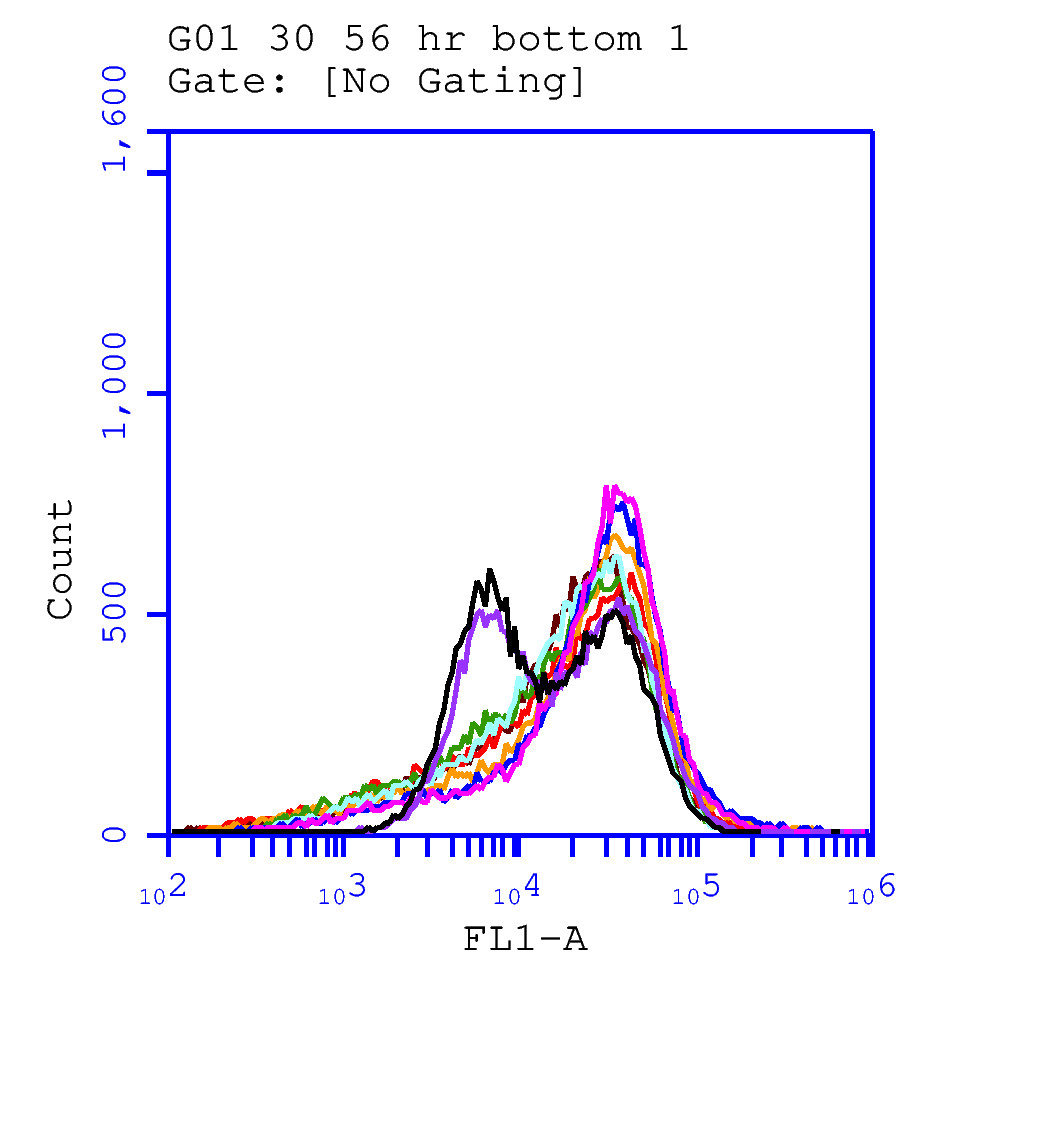

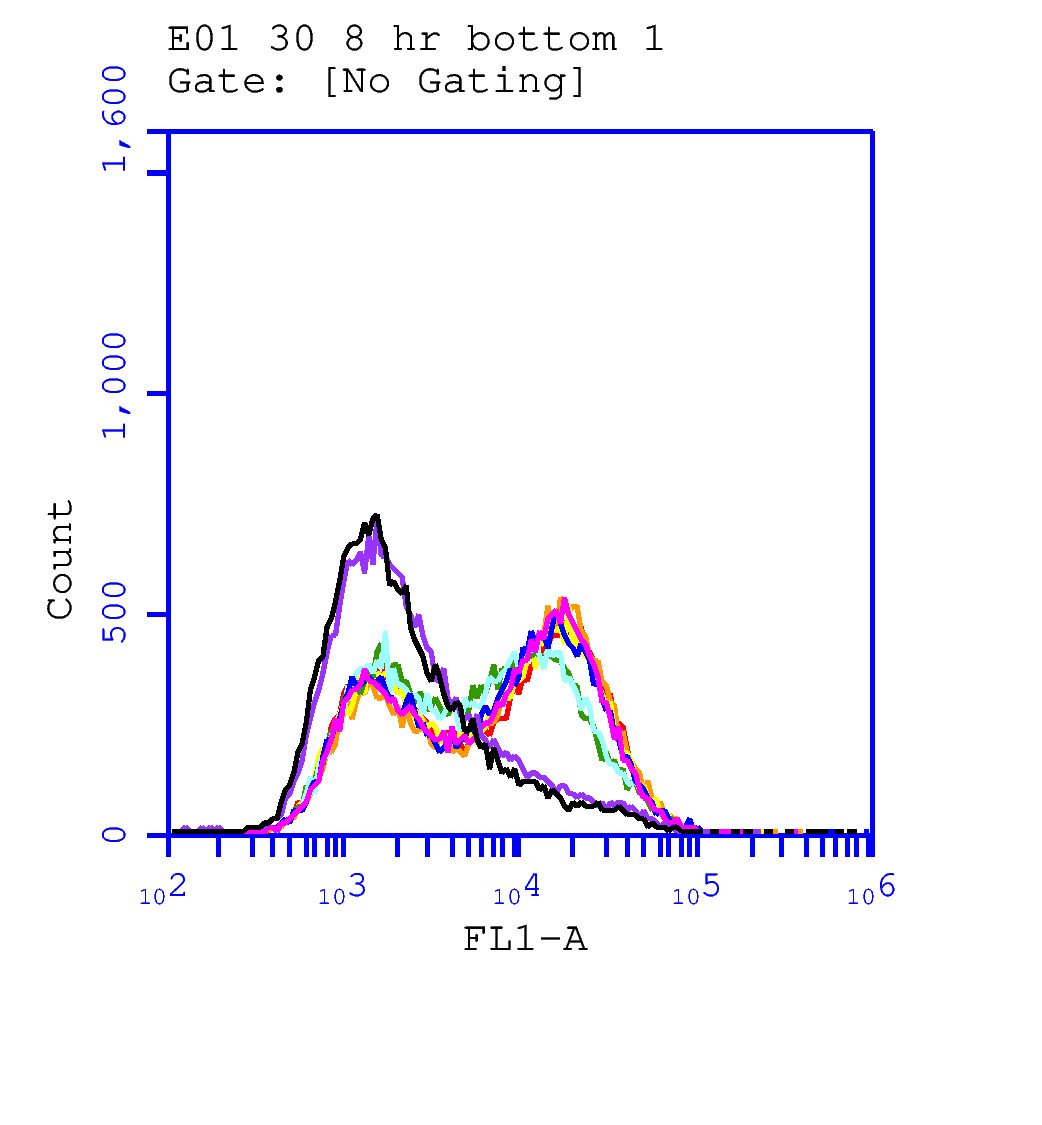

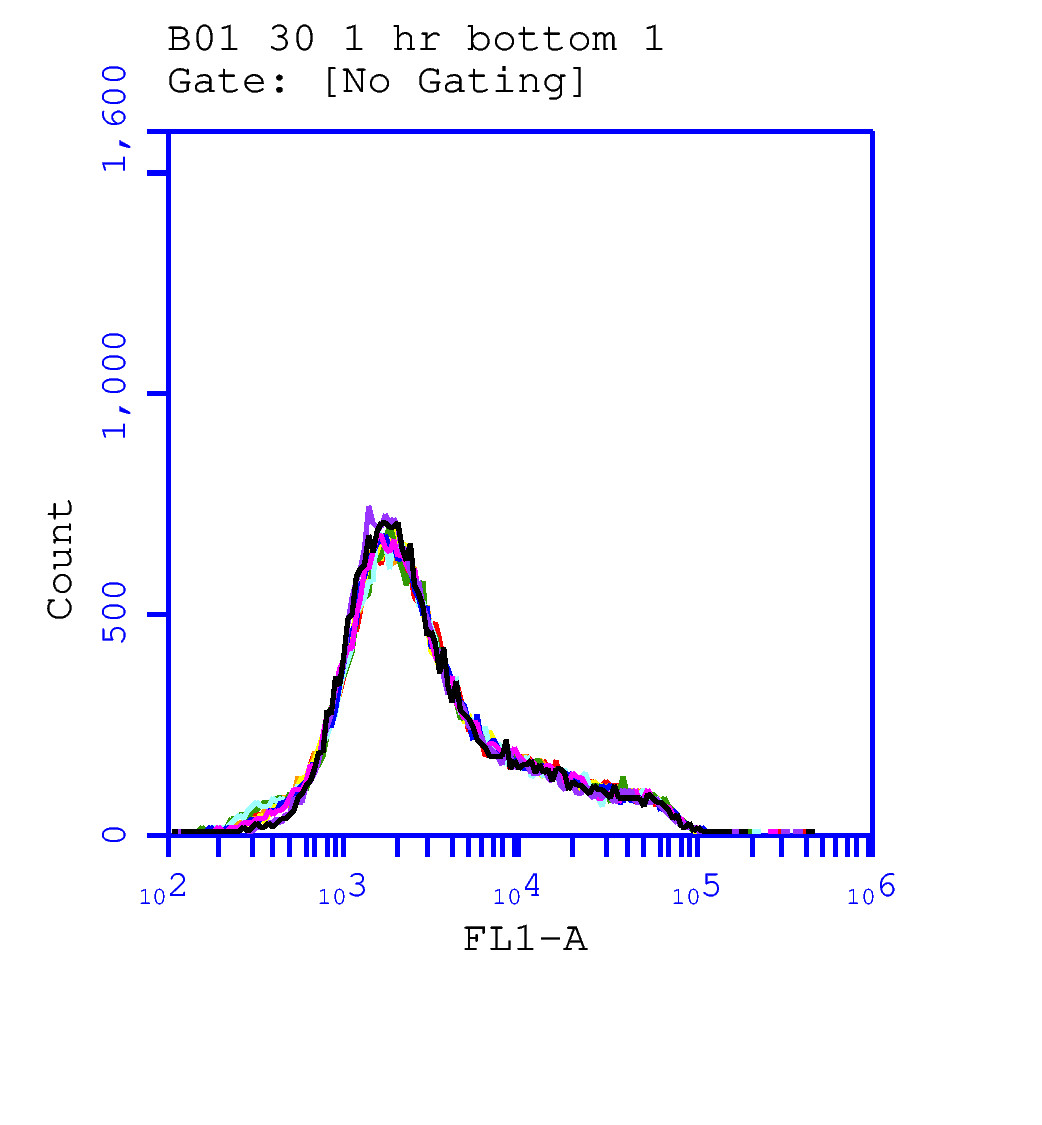

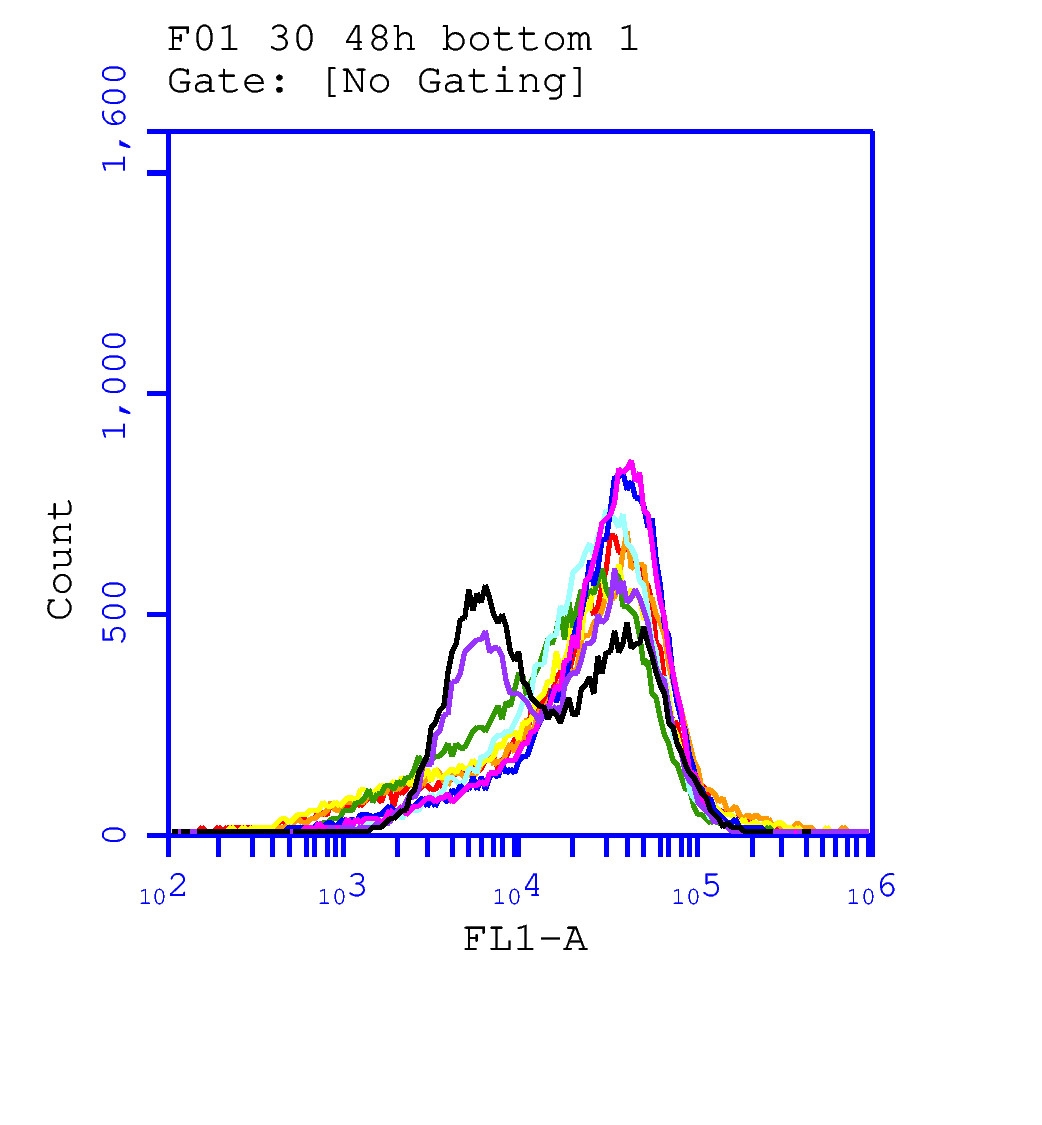

Supplement: Supplementary file 1 — Supplementary file1 (DOCX 4563 kb) [file 203_2020_1864_MOESM1_ESM.docx]
